# Identification of Key Active‐Site Positions Controlling the Chemoselectivity of *Aspergillus Brasiliensis* Unspecific Peroxygenase

**DOI:** 10.1002/cbic.202500181

**Published:** 2025-05-06

**Authors:** Fabian Schmitz, Maike Hoffrogge, Katja Koschorreck, Yasuhisa Fukuta, Alessandra Raffaele, Florian Tieves, Thomas Hilberath, Frank Hollmann, Vlada B. Urlacher

**Affiliations:** ^1^ Institute of Biochemistry Heinrich‐Heine‐Universität Universitätsstraße 1 40225 Düsseldorf Germany; ^2^ Faculty of Agriculture Kindai University 3327‐204 Naka‐machi Nara 631‐8505 Japan; ^3^ Department of Biotechnology Delft University of Technology Van der Maasweg 9 2629 Hz Delft The Netherlands

**Keywords:** aromatic hydroxylations, biocatalyses, chemoselectivities, protein engineerings, unspecific peroxygenases

## Abstract

Heme‐containing unspecific peroxygenases (UPOs) have attracted significant attention as biocatalysts for oxidation reactions due to their ability to function without expensive nicotinamide cofactors. In the recent study, the UPO from *aspergillus brasiliensis* (*Abr*UPO) is found to catalyze the aromatic hydroxylation of substituted benzenes, a feature that distinguishes *Abr*UPO from other reported wild‐type UPOs. To elucidate the underlying factors in the active site and substrate access channel of *Abr*UPO—which contains fewer phenylalanine residues compared to other UPOs that primarily catalyze benzylic hydroxylation—twenty two *Abr*UPO variants with single, double, triple, or quadruple amino acid substitutions were constructed to mimic the active sites or substrate access channels of other UPOs. A number of mutated variants exhibited altered activity and selectivity, and several positions were identified that influence enzyme chemoselectivity. Among them, substitution of alanine at position 186 with bulkier residues such as phenylalanine or leucine lead to a shift in chemoselectivity toward alkyl chain hydroxylation of substituted benzenes. Molecular docking studies indicated that the A186F mutation restricts the flexibility and reorientation of ethylbenzene in the active site of *Abr*UPO, thereby preventing oxidation at the aromatic ring while promoting benzylic hydroxylation.

## Graphical abstract

Unspecific peroxygenase (UPO) from *Aspergillus brasiliensis* *Abr*UPO catalyzes the aromatic hydroxylation of substituted benzenes. By integrating structural elements from well‐characterized UPOs in *Abr*UPO variants, position 186 was identified as a critical determinant of enzyme chemoselectivity. The A186F substitution prevents aromatic hydroxylation of substituted benzenes in favor of benzylic hydroxylation.

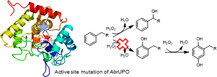

## 1 Introduction

Fungal unspecific peroxygenases (UPOs) (EC 1.11.2.1) are heme‐thiolate enzymes that follow the catalytic mechanism of classical peroxidases and the peroxide shunt pathway of P450 monooxygenases (P450s).^[^ ^1^ ^]^ According to a phylogenetic analysis, UPOs have been divided into two distinct families, each characterized by unique structural traits and substrate preferences. Family I comprises “short” UPOs, while Family II consists of “long” UPOs.^[^ ^1^ ^]^ Utilizing hydrogen peroxide as co‐substrate, UPOs can either oxidatively dehydrogenate a substrate (peroxidase activity) or insert an oxygen atom into nonactivated C—H bonds (peroxygenase activity). The reactions catalyzed by UPOs include hydroxylation of aromatic and aliphatic substrates, epoxidation of alkenes, O‐/N dealkylation, N‐/S oxidation, and halogenation.^[^ ^2^ ^]^ Due to this broad spectrum of catalyzed oxidation reaction, UPOs have garnered significant interest in industrial and environmental biocatalysis.^[^ ^3^ ^]^ During biocatalysis with UPOs, overoxidation of the formed alcohols to aldehydes/ketones and acids can occur.^[^ ^3a^ ^]^ In certain cases, this property can be advantageous for the formation of valuable acid products.^[^ ^4^ ^]^ More often, such overoxidation is undesirable, and the factors contributing to it, such as oxidative dehydrogenation of the formed alcohol product via peroxidase activity of UPO, are currently under investigation.

Aromatic hydroxylation of substituted benzenes is of particular interest, as the resulting phenolic products play an important role in daily life, serving as key building blocks in the synthesis of dyes, pharmaceuticals, and agrochemicals.^[^ ^5^ ^]^ Enzyme‐catalyzed aromatic hydroxylation offers a promising alternative to traditional chemical methods, which often face challenges with low efficiency or selectivity during aromatic oxidation.^[^ ^6^ ^]^

While some P450s have been reported to catalyze selective aromatic oxidation of substituted benzenes, this activity is rarely described for UPOs.^[^ ^7^ ^]^ For instance, the evolved variant of *Agrocybe aegerita* UPO, PaDa‐I—the most extensively studied UPO catalyzes the hydroxylation of naphthalene, benzene, and toluene at the aromatic ring, but it failed to catalyze aromatic hydroxylation of substituted benzenes like ethyl‐ or propylbenzene.^[^ ^2^, ^8^ ^]^ Only trace amounts of aromatic hydroxylation products were observed with PaDa‐I in the oxidation of butylbenzene, isobutylbenzene, and *sec*‐butylbenzene.^[^ ^9^ ^]^ It has been proposed that UPOs facilitate aromatic hydroxylation by utilizing the highly active Compound I, which attacks the π‐system of the aromatic ring, forming an arene oxide intermediate that can spontaneously rearrange to the corresponding phenol (NIH shift).^[^ ^1^, ^2^, ^10^ ^]^ This mechanism is analogous to that of P450s, where an electrophilic and/or radical pathway results in aromatic hydroxylation.^[^ ^11^ ^]^

Recently, we characterized a “short” UPO from *Aspergillus brasiliensis* (*Abr*UPO) that catalyzes aromatic hydroxylation of substituted benzenes and phenols with various branched or unbranched alkyl chains.^[^ ^9^, ^12^ ^]^ For instance, with ethylbenzene as substrate, *Abr*UPO leads to both aromatic ring and benzylic hydroxylation products in a 1:1 ratio.^[^ ^9^ ^]^ We also observed that the efficiency of aromatic hydroxylation strongly depends on the length and type of the alkyl chain, with activity decreasing as the chain length increases.

**Figure 1:**
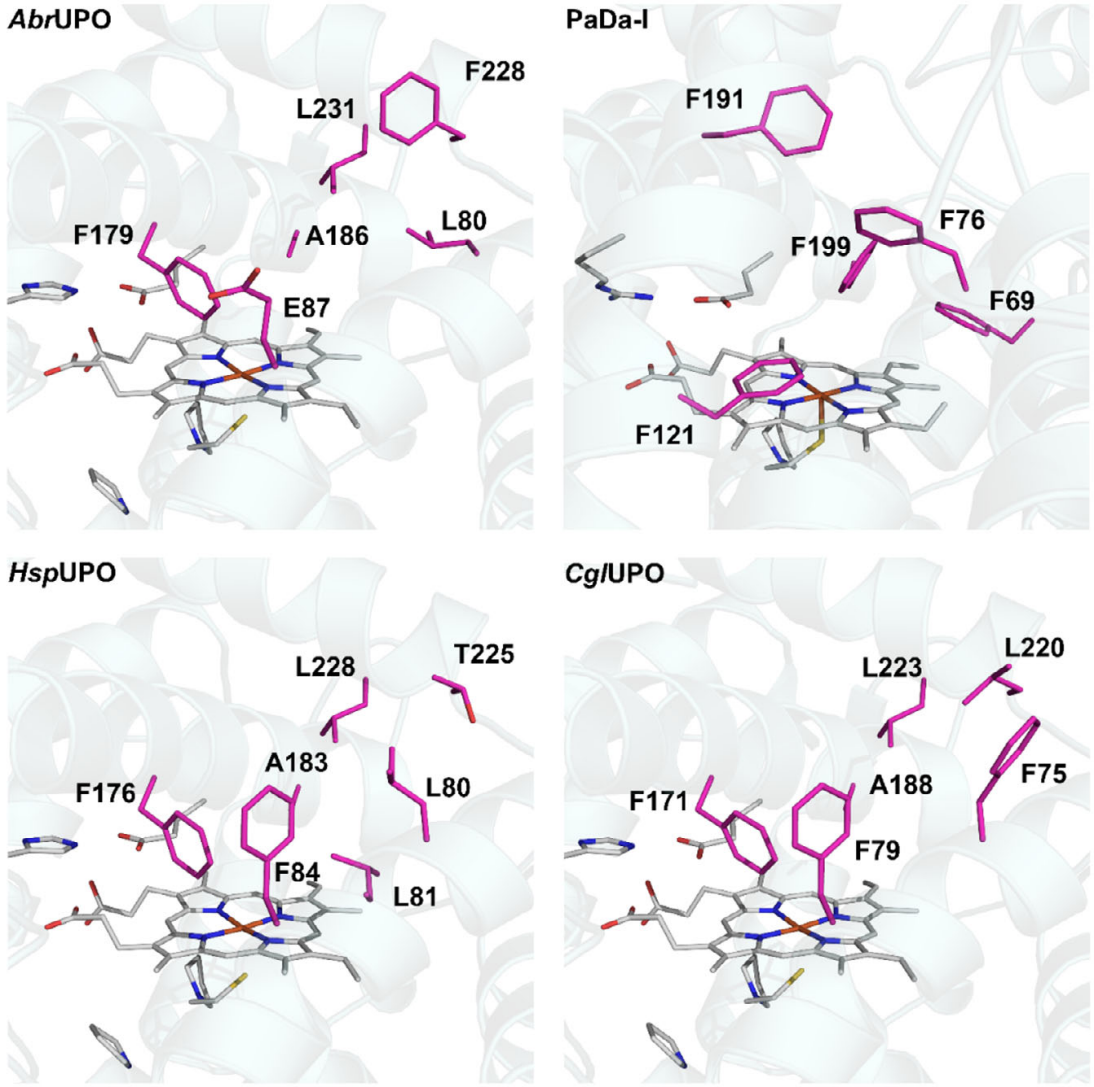
Residues located in the active site and substrate access channel of *Abr*UPO, PaDa‐I (5OXU), *Hsp*UPO (7O1X), and *Cg*lUPO. Homology models for *Abr*UPO and *Cgl*UPO were obtained using AlphaFold 2.

A structural comparison of *Abr*UPO with three well‐studied UPOs, PaDa‐I, *Cgl*UPO from *Chaetomium globosum*, and *Hsp*UPO from *Hypoxylon* sp. revealed several structural differences within *Abr*UPO's active site and substrate access channel (**Figure** **1**). Notably, *Abr*UPO has only one phenylalanine residue positioned close to the heme, whereas PaDa‐I contains a triad of phenylalanine residues (F69, F121, F199) that orients aromatic substrates through π–π stacking toward the heme group. This structural feature in PaDa‐I may prevent the oxidation of substituted benzenes like ethylbenzene at the aromatic ring.^[^ ^9^, ^13^ ^]^

To gain a deeper understanding of the effect of individual residues in the active site and substrate access channel on the chemoselectivity of *Abr*UPO in the oxidation of substituted benzenes, we constructed and studied 22 *Abr*UPO variants, each containing one, two, three, or four mutations. Our results suggest that while several positions influence the product pattern, position 186 plays a crucial role in modulating the chemoselectivity of *Abr*UPO.

## 2 Results and Discussion

### 2.1 Construction of *Abr*UPO Active‐Site Variants

The structural comparison of the active site of *Abr*UPO with well‐characterized UPOs, the “long” UPO PaDa‐I and two “short” UPOs *Cgl*UPO and *Hsp*UPO, revealed that *Abr*UPO has fewer phenylalanine residues within its substrate‐binding site and substrate access channel (Figure 1).^[^ ^14^ ^]^ The *Abr*UPO active site possesses only one phenylalanine residue, F179, near the heme group, which is also found in the structurally similar *Hsp*UPO (F176) and *Cgl*UPO (F171). In contrast, the positions corresponding to F69 and F199 in PaDa‐I are occupied by L80 and A186 in *Abr*UPO. Additionally, *Abr*UPO features a polar glutamic acid residue at position 87, whereas *Hsp*UPO and *Cgl*UPO have a phenylalanine at the homologous position (F84 in *Hsp*UPO and F79 in *Cgl*UPO). Another notable difference is the presence of a phenylalanine residue (F228) at the entrance of the substrate access channel in *Abr*UPO, whereas this position is occupied by threonine in *Hsp*UPO (T225) and leucine in *Cgl*UPO (L220).^[^ ^9^, ^14^ ^]^ Furthermore, *Abr*UPO contains a leucine residue (L231) opposite F228, which may potentially restrict the substrate access to the heme; this residue is also present in *Hsp*UPO (L228) and *Cgl*UPO (L223).^[^ ^9^ ^]^

To further elucidate *Abr*UPO's capability to catalyze the aromatic hydroxylation of substituted benzenes, we targeted positions L80, E87, F179, A186, F228, and L231 for site‐directed mutagenesis. We introduced phenylalanine residues at positions 80, 87, and 186 to create the *Abr*UPO variants L80F, E87F, and A186F, respectively, mimicking the active sites of *Cgl*UPO, *Hsp*UPO, and PaDa‐I (Figure 1).^[^ ^14^ ^]^ Additionally, we constructed the double‐mutant L80F_A186F to replicate the phenylalanine triad of PaDa‐I and investigate its impact on the chemoselectivity.^[^ ^2^, ^15^ ^]^ Beyond A186F, we also examined the effect of introducing smaller, less hydrophobic amino acids by creating the A186G (glycine) and A186L (leucine) variants. To explore the role of the conserved phenylalanine residue F179 in substrate oxidation, we replaced it with alanine (F179A) or leucine (F179L). Given that the substrate access channel in *Abr*UPO appears to be restricted by F228 and L231, we substituted F228 with leucine (F228L), as found in *Cgl*UPO, and L231 with alanine (L231A) to widen the entrance of the substrate access channel for bulkier substrates. We also combined several single mutations to create double, triple, and quadruple mutants (**Table** **1**). All *Abr*UPO variants, except for F228L_L231A, were successfully expressed in *Komagataella phaffii* under control of the AOX1 promoter. The α‐factor secretion signal peptide from *Saccharomyces cerevisiae* was employed for extracellular expression, as it yielded the highest extracellular activity of the wild‐type (WT) enzyme compared to other secretion signal peptides tested (Figure S1, Supporting Information). We initially investigated whether the introduced mutations affected expression as well as specific peroxidase or peroxygenase activity, given that mutations in the active site or substrate access channel are known to influence both UPO activities.^[^ ^13^, ^16^ ^]^ For instance, Molina‐Espeja et al. demonstrated that mutations in the access channel of PaDa‐I influenced its peroxidase activity and reduced the possibility of a long‐range electron transfer pathway from oxidizable residues like tyrosine on the protein surface to the heme, as seen in ligninolytic peroxidases.^[^ ^17^ ^]^

**Table 1:** Volumetric activity, specific peroxidase activity, specific peroxygenase activity, and the peroxygenase:peroxidase (P:p) ratio of *Abr*UPO WT and single, double, triple, or quadruple mutants. Volumetric activity serves as indicator of UPO expression and was determined with cell‐free culture supernatant.

*Abr*UPO variant	Volumetric activities ABTS [U ml^−1^]	Specific activities NBD [P] [U mg^−1^]	Specific activities ABTS [p] [U mg^−1^]	P:p ratio
WT	30.9	2.1	2.1	1.0
L80F	42.1	6.3	11.1	0.6
E87F	46.4	7.7	9.7	0.8
F179A	48.4	9.1	33.1	0.3
F179L	19.9	9.3	8.9	1.0
A186G	16.8	50.5	27.3	1.8
A186L	12.8	7.7	4.5	1.7
A186F	69.9	9.8	14.9	0.7
F228L	22.1	8.5	4.6	1.8
L231A	3.4	15.1	0.9	16.7
L80F_A186F	24.3	1.2	2.0	0.6
L80F_F228L	17.0	10.0	1.9	5.3
L80F_L231A	6.2	18.3	1.1	16.6
F179A_A186F	7.3	0.5	1.2	0.4
F179L_A186F	31.5	0.8	4.8	0.2
A186F_F228L	84.4	3.9	17.2	0.2
A186F_L231A	39.5	3.9	8.1	0.5
F228L_L231A	/	/		/
L80F_A186F_F228L	146.4	8.4	25.7	0.3
L80F_A186F_L231A	55.5	4.7	10.3	0.5
L80F_F228L_L231A	30.8	13.8	4.4	3.1
A186F_F228L_L231A	29.2	3.4	5.7	0.6
L80F_A186F_F228L_L231A	61.7	10.2	13.7	0.7

5‐Nitro‐1,3‐benzodioxole (NBD) was used as a peroxygenase substrate and 2,2'‐azino‐bis(3‐ethylbenzothiazoline‐6‐sulfonic acid) (ABTS) as a peroxidase substrate. The variants L80F, E87A, and F179A and particularly A186F, A186F_F228L, L80F_A186F_F228L, and L80F_A186F_F228L_L231A demonstrated higher volumetric activities (measured with ABTS) than *Abr*UPO WT enzyme (*Abr*UPO WT). As shown in Table 1, this is not only due to higher specific activity of these variants but also due to enhanced expression, especially for the A186F_F228L variant. The A186G mutation induced a strong increase in peroxidase activity, although not as strong as in peroxygenase activity. Specific peroxygenase activity with NBD increased 3–25‐fold for almost all single mutants, with the A186G variant exhibiting specific activity of 50 U mg^−1^ (vs 2 U mg^−1^ for WT). In general, all single mutants showed increased activity, whereas the activity of the double mutants was unequally affected by changes in the active center.

Specific activities with NBD (P) and with ABTS (p) were used to determine the ratio of peroxygenase to peroxidase activity for *Abr*UPO variants (designated as the P:p ratio in Table 1).

Compared to *Abr*UPO WT, the P:p ratio changed for several variants either toward peroxygenase or toward peroxidase activity. Particularly, the L231A mutation switched toward peroxygenase with an almost 17‐fold higher P:p ratio for the single mutant L231A and for the double‐mutant L80F_L231A (Table 1). This mutation, introduced to broaden the substrate access channel, may have facilitated NBD access to the heme while simultaneously reducing peroxidase activity. Notably, the mutation L231A negatively affected expression of both variants. The double‐mutant F228L_L231A, with both mutations located at the entrance of the access channel, could not be functionally expressed. The PaDa‐I‐derived double‐mutant JaWa, with the mutation G241D, located in the substrate access channel, and the R257K, located at the protein's surface, was also reported to have improved peroxygenase activity, while peroxidase activity was reduced.^[^ ^16^ ^]^

### 2.2 Influence of Active‐Site Mutations on Aromatic Hydroxylation

Further, we investigated the impact of the introduced mutations on activity and chemoselectivity of *Abr*UPO with ethylbenzene (**1a**), propylbenzene (**2a**), butylbenzene (**3a**), cumene (**4a**), and *p*‐cymene (**5a**) as substrates (**Table** **2**). The substrate conversion and the ratio of aromatic to nonaromatic oxidation products formed during conversion of **1a**–**5a** by the *Abr*UPO variants is presented in **Table** **3**. Aromatic hydroxylation catalyzed by *Abr*UPO WT decreased from 60% for **1a** to 30% for **2a** and 9% for **3a,** resulting in aromatic:nonaromatic ratios of 1.5, 0.4, and 0.1, respectively (Table 3 and Figure S2–S6, Supporting Information).^[^ ^9^ ^]^ Similar to *Abr*UPO WT, for most of the single mutants, the proportion of aromatic hydroxylation decreased as the length or branching of the alkyl chain increased from **1a** to **3a** or **4a**. The most significant effect on the aromatic hydroxylation of the substrates tested was observed in variants with substitutions at position 186, located in the α‐helix above the heme group. Only trace amounts of aromatic hydroxylation products were detected in reactions with **1a**–**4a** catalyzed by the A186L and A186F variants, while their activity was comparable to that of WT and the A186G variant. Reactions catalyzed by the A186G variant gave fewer aromatic hydroxylation products of **1a** but yielded more of **2a** and **3a** compared to the WT. The presence of glycine at position 186 may provide additional space within the active site, allowing for altered orientation of bulkier substrates. We are currently investigating why the amount of aromatic hydroxylation product(s) of **1a** is so low with the A186G mutant. With **5a**, the A186L mutation had a negative effect on enzyme activity resulting in 8% conversion, whereas with WT and the A186G conversion achieved over 70%. The A186Y mutation also resulted in reduced activity with **5a** (6% conversion), while variants with valine or isoleucine at this position resulted in 27% conversion. Notably, cumin alcohol (**5g**) was the main product formed during **5a** conversion by the A186V, A186L, A186I, and A186Y variants (Figure S6 and S7, Supporting Information). The A186F mutation completely prevented the **5a** oxidation (Table 3). In all double, triple, or quadruple mutants, the bulky F186 also either prevented aromatic hydroxylation or resulted in activity loss (Table 3). For instance, the L80F_A186F variant, designed to mimic the phenylalanine triad of PaDa‐I, showed no aromatic hydroxylation with **1a**–**5a**, similar to PaDa‐I (Figure S2–S6, Supporting Information).^[^ ^9^ ^]^

**Table 2:** Investigated substituted benzenes **1a**–**5a** and detected oxidation products.

Substrate	Benzylic oxidation products	Aromatic oxidation products
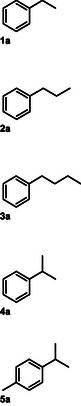	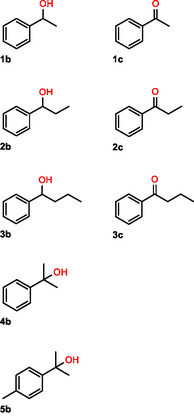	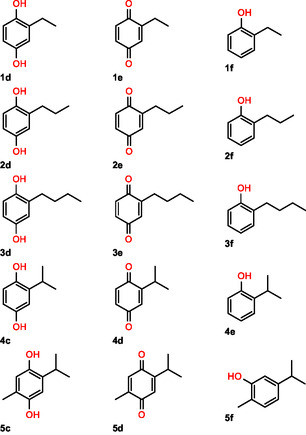

**Table 3:** Conversion of **1a**–**5a** with *Abr*UPO WT and variants.

Substrate conversion [%] (aromatic:nonaromatic oxidation ratio) for each substratea)					
***Abr*** **UPO variant**	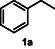	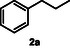	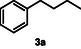	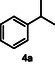	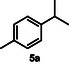
Wild type	45 (1.5)	78 (0.4)	64 (0.1)	53 (0.2)	72 (1.2)
L80F	40 (0.9)	88 (0.5)	35 (0.1)	39 (0.3)	67 (1.0)
E87F	34 (0.6)	86 (0.3)	70 (0.1)	62 (0.1)	30 (0.8)
F179A	9 (0.9)	54 (0.7)	30 (0.5)	22 (−)	16 (0.8)
F179L	7 (1.3)	48 (1.3)	40 (0.9)	20 (0.1)	25 (0.3)
A186G	34 (0.3)	80 (0.6)	47 (0.5)	40 (0.2)	71 (0.6)
A186L	22 (−)	68 (−)	58 (−)	34 (0.1)	8 (−)
A186F	32 (−)	88 (−)	75 (−)	64 (0.1)	/
F228L	43 (1.2)	77 (0.5)	61 (0.3)	63 (0.3)	80 (1.5)
L231A	64 (0.4)	75 (0.3)	59 (0.2)	73 (0.1)	78 (0.7)
L80F_A186F	40 (−)	79 (−)	60 (−)	62 (−)	/
L80F_F228L	35 (0.8)	82 (0.5)	30 (0.1)	39 (0.5)	65 (2.3)
L80F_L231A	40 (0.1)	88 (0.2)	60 (0.1)	72 (0.1)	83 (0.3)
F179A_A186F	17 (−)	59 (−)	60 (−)	68 (−)	/
F179L_A186F	/	/	/	/	/
A186F_F228L	25 (−)	92 (−)	74 (−)	58 (−)	/
A186F_L231A	41 (0.1)	86 (0.1)	39 (−)	91 (−)	/
L80F_A186F_F228L	35 (−)	86 (−)	62 (−)	70 (−)	/
L80F_A186F_L231A	43 (−)	73 (−)	33 (−)	39 (−)	27 (−)
L80F_F228L_L231A	51 (0.4)	71 (0.4)	27 (0.1)	21 (0.2)	34 (1.5)
A186F_F228L_L231A	18 (−)	88 (−)	50 (−)	75 (−)	/
L80F_A186F_F228L_L231A	13 (−)	81 (−)	40 (−)	53 (−)	/

a) /means no conversion was observed; (−) means only low amounts of aromatic oxidation products (3%–6%) were observed giving ratios of ≤ 0.1.

Interestingly, recently, some computationally designed enantio‐divergent PaDa‐I variants, carrying 4–5 mutations in the active site, were shown to catalyze aromatic hydroxylation of **1a** at *ortho* or *para* position.^[2a]^ All those PaDa‐I variants carried the mutation F199L (corresponding to A186L in *Abr*UPO) and some of them additionally the F69L substitution (corresponding to L80 in *Abr*UPO). These results support our findings that position 186 plays a crucial role not only in chemoselectivity but also substrate acceptance in *Abr*UPO.

The introduction of phenylalanine at positions 80 (L80F) or 87 (E87F) in *Abr*UPO also reduced the formation of aromatic hydroxylation products, particularly in reactions with **1a** and **5a**. Therefore, both positions appear to play a crucial role in the chemoselectivity of *Abr*UPO during the hydroxylation of the compounds investigated; however, the effect was not as pronounced as with the A186L and A186F mutations. Position 179 is located above the heme. The F179A and F179L variants demonstrated significantly lower conversions of **1a**–**5a** than *Abr*UPO WT (Table 3). In contrast, the percentage of aromatic hydroxylation products in reactions with **2a** and **3a** was higher. Notably, while the WT enzyme partially oxidized **5a** to carvacrol (**5f**) and further to the main product thymohydroquinone (**5c**), the F179L variant catalyzed the reaction only to carvacrol (**5f**). Instead, this variant also catalyzed the benzylic hydroxylation of **5a**, producing cumin aldehyde (**5h**) as one of the three main products (Figure S6, Supporting Information), similar to PaDa‐I. Thus, position 179 in *Abr*UPO appears to influence both activity and chemoselectivity.

The replacement of the phenylalanine at position 228 at the entrance of the substrate access channel with the smaller leucine (F228L), similar to *Cgl*UPO, had only a marginal effect on enzyme chemoselectivity with **1a**–**4a**. However, combining the F228L and L80F mutations led to increased aromatic hydroxylation during **5a** conversion (70% vs 50% with WT) (Table 3, Figure S6, Supporting Information). These results demonstrate that changes in enzyme selectivity depend on both the introduced mutation(s) and the specific substrate used, a phenomenon also observed for PaDa‐I mutants.^[15a]^

Interestingly, the L231A variant, designed to broaden the substrate access channel, exhibited a shift in regioselectivity during the hydroxylation of **3a** at the alkyl chain. While *Abr*UPO WT primarily oxidized at the benzylic position, producing alcohol **3b** and ketone **3c** as the main products, the L231A variant also oxidized the alkyl chain at the ω‐1 position, yielding 4‐phenyl‐2‐butanone (**3h**) along with **3b** and **3c** in similar ratios (Figure S4, Supporting Information). In contrast, PaDa‐I failed to oxidize **3a** at the benzylic position, resulting in 4‐phenyl‐2‐butanol (**3g**) as the main product.^[^ ^9^ ^]^ The absence of **3g** in the reaction with the L231A variant and the exclusive formation of **3h** might be attributed to the increased peroxygenase activity of this variant, as measured with NBD (Table 1). The formation of ketones from benzylic alcohols has been shown to depend on both the peroxidase and peroxygenase activities of *Abr*UPO, with the latter being primarily responsible when using 1‐phenyl‐1‐butanol (**3b**) as a substrate.^[^ ^9^ ^]^ Our results indicate that position 231, although located at the substrate access channel, plays a role in modulating the regioselectivity of oxidation in longer‐chain alkyl benzenes. Similarly, a slight enlargement of the substrate access channel in *Cvi*UPO from *Collariella virescens* via the F88L mutation was shown to affect the selectivity of linoleic acid oxidation.^[^ ^18^ ^]^

### 2.3 Oxidation of Substituted Benzenes and Phenols by the A186F Mutant

Since the A186F mutation had the most significant impact on the chemoselectivity of *Abr*UPO, we studied this variant in more detail. The A186F variant was produced in a fed‐batch fermentation (Figure S8, Supporting Information), purified via hydrophobic interaction chromatography (HIC), and subsequently applied in reactions with ≈7 mM substrates **1a**–**3a**. The results from previous experiments with lower substrate concentrations were confirmed (Table S1, Supporting Information). While *Abr*UPO WT produced up to 57% aromatic hydroxylation products, only trace amounts (1%–3%) of aromatic hydroxylation products were detected in reactions catalyzed by the A186F variant (Table S1, Supporting Information). Correspondingly, the amount of benzylic oxidation products increased. In these reactions, overoxidation of alcohols to ketones increased with increasing length of the alkyl chain, similar to the WT enzyme. The introduced mutation also slightly enhanced enantioselectivity for the oxidation of 1‐phenyl‐1‐butanol (**3b**) (Table S1, Supporting Information).

Additionally, we selected several *ortho*‐substituted monophenols (2‐ethylphenol **1f**, 2‐isopropylphenol **3f**, and thymol **5e**) to investigate whether the introduced mutation also affects the oxidation of phenols. Although the A186F variant was almost incapable of oxidizing the substituted benzenes **1a**–**5a** at the aromatic ring, it enabled aromatic hydroxylation of **1f**, **3f**, and **5e** to the corresponding hydroquinones. The conversion of **1f** and **3f** was comparable to that of the WT,^[^ ^12^ ^]^ while **5e** was oxidized to a much lower extent (**Table** **4**). These results suggest that the aromatic hydroxylation of nonactivated aromatic substrates is strongly influenced by geometric constraints within the active site of *Abr*UPO, whereas the oxidation of phenolic substrates is less affected by these factors.

**Table 4:** Hydroxylation of phenols catalyzed by *Abr*UPO WT and variant A186F.

Substrate	Substrate conversion [%]	
	WT	A186F
2‐Ethylphenol **1f**	97	90
2‐Isopropylphenol **3f**	85	74
Thymol **5e**	99	31

### 2.4 Molecular Docking

**Figure 2:**
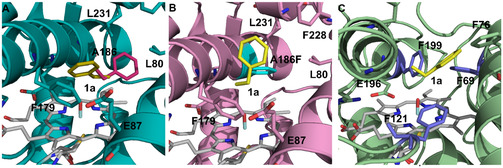
Molecular docking analysis for ethylbenzene **1a** (yellow) with A) *Abr*UPO (teal), B) A186F (rose), and C) PaDa‐I (green). For *Abr*UPO, two possible poses of **1a** are shown, and pose two is highlighted in pink. Mutation A186F is shown in cyan. The phenylalanine triad (F69, F121, and F199) of PaDa‐I is highlighted in blue.

To rationalize the results concerning the impact of position 186 on the chemoselectivity of *Abr*UPO in the oxidation of substituted benzenes, molecular docking was conducted on the WT enzyme and A186F variant with **1a**. PaDa‐I was used for comparison (**Figure** **2**).

Molecular docking analysis revealed that **1a** has sufficient space in the active site of *Abr*UPO WT to adopt an unrestricted orientation, resulting in two possible poses (Figure 2A, yellow and pink poses). Both benzylic and the aromatic positions are at a close distance of 3 Å from the heme iron (yellow pose). This supports our experimental results, which showed the formation of both aromatic and benzylic hydroxylation products during **1a** oxidation.

However, this unrestricted orientation is significantly constrained in the A186F variant (Figure 2B), as the active site is considerably smaller due to the introduced phenylalanine residue. Consequently, only one possible pose for **1a** is feasible. The steric hindrance caused by the introduced phenylalanine residue limits the substrate's ability to adopt various poses, leading to exclusive hydroxylation at the benzylic position, as confirmed by our experimental results. It is possible that substrate movement may be further restricted by π–π stacking interactions with the introduced phenylalanine residue.

In the active site of PaDa‐I, the phenylalanine triad plays a crucial role in the binding and orientation of aromatic substrates through π–π stacking, which could restrict substrate movement.^[15c]^ Docking simulations revealed that in PaDa‐I, **1a** is oriented with its alkyl side chain at a catalytic distance of 3.74 Å from the heme iron (Figure 2C), which supports the observed absence of aromatic hydroxylation products. The significant influence of phenylalanine residues in the active site of UPOs on aromatic hydroxylation was also demonstrated in variants of *Mth*UPO from *Myceliophthora thermophila*. Experimental data, molecular docking, and molecular dynamics simulations revealed that in the L60F variant, 2‐methylnaphthalene is positioned with its methyl group near the heme, leading to aliphatic hydroxylation. In contrast, in the triple variant L60F‐A161F‐S159G, the substrate is oriented for aromatic hydroxylation.^[^ ^19^ ^]^ Coleman et al. also showed that the geometric constraints at the active site of P450s can drastically influence the ratio between aromatic and aliphatic hydroxylation.^[7b,c]^

Additionally, “long” UPOs, such as PaDa‐I tend to have longer and more buried substrate access channels compared to “short” UPOs.^[1a]^ This also might result in a more restricted substrate orientation in the active site.

## 3 Conclusion

The WT *Abr*UPO was found to catalyze the aromatic hydroxylation of substituted benzenes. Depending on the structure of the substrates tested, it can also catalyze the hydroxylation at the benzylic position or other positions on the alkyl side chain. To investigate the influence of different active‐site positions and amino acids on the chemoselectivity of *Abr*UPO, we created a set of single mutants and several combinations thereof. It is noteworthy that the structural modifications introduced in *Abr*UPO to emulate the PaDa‐I active site have also resulted in alterations in chemoselectivity toward that of PaDa‐I, which confirm the importance of this approach for structural studies and enzyme engineering.

Our results revealed that amino acids at positions 80, 87, 179, and 186 in the active site of *Abr*UPO affect the ratio between aromatic and alkyl chain hydroxylation of the substituted benzenes tested. Among them, position 186 is the key determinant controlling the enzyme chemoselectivity. While the presence of phenylalanine at position 186 prevented aromatic hydroxylation, and shifted the chemoselectivity toward alkyl chain hydroxylation, glycine at position 186 supported aromatic hydroxylation. These findings provide a solid foundation for further mutational studies aiming at tailoring the chemoselectivity and activity of UPOs.

## 4. Experimental Section

1

1.1

##### Strains

*Escherichia coli* DH5α (Clontech Laboratories Inc., Heidelberg, Germany) was used for cloning procedures. *Komagataella phaffii* (formerly known as *Pichia pastoris* X‐33) was used for expression of UPO genes, purchased from Invitrogen (Carlsbad, USA).

##### Cloning and Site‐Directed Mutagenesis

The plasmid pPICZαA_af‐*Abr*UPO containing the α‐factor pre–pro sequence for *Abr*UPO secretion was used as template for site‐directed mutagenesis using a two‐step QuikChange protocol with oligonucleotides listed in Table S2, Supporting Information. pPICZαA_af‐*Abr*UPO was constructed from pPICZA_*Abr*UPO using primers αf‐*Abr*UPO_fw and pPICZA_rev (Table S2, Supporting Information) for *Abr*UPO sequence amplification. The product of polymerase chain reaction (PCR) was digested with *Xho*I and *Not*I and ligated into the corresponding restriction sites of pPICZαA. Chemically competent *E. coli*DH5α cells were transformed with the ligation product and plated onto selective luria‐bertani (LB) agar plates (10 g l^−1^ peptone, 5 g l^−1^ yeast extract, 5 g l^−1^ NaCl, 15 g l^−1^ agar) containing 25 μg ml^−1^ Zeocin (InvivoGen, San Diego, USA).

Plasmid isolation was carried out using the ZR Plasmid Miniprep Kit (Zymo Research, Freiburg, Germany) according to the manufacturer's instructions. Introduced DNA modifications were verified through DNA sequencing by Eurofins Genomics Germany GmbH (Ebersberg, Germany). For site‐directed mutagenesis, 50–100 ng of pPICZαA_af‐*Abr*UPO was mixed with 200 nM either forward and reverse primer, 200 μM of each dNTP, 1 × high‐fidelity buffer, 3% dimethyl sulfoxide, and 0.02 U μL^−1^ Phusion High‐Fidelity DNA polymerase (Thermo Fisher Scientific, Bremen, Germany) in a total volume of 25 μl. The following PCR protocol was used for amplification of the individual DNA strands: initial denaturation at 98 °C for 30 s, 5 times cycling of denaturation at 98 °C for 30 s, annealing at 62–70 °C for 45 s, and extension at 72 °C for 210 s, followed by a final extension at 72 °C for 7 min.

##### Expression of AbrUPO WT and Variants

For prescreening, up to fourty *K. phaffii* colonies for each variant were selected from yeast extract peptone dextrose sorbitol (YPDS)‐agar plates and grown in 200 μl buffered glycerol‐complex medium with yeast extract (BMGY) in 96‐deep well plates (MegaBlock96 well, 1.2 mL, Sarstedt, Nümbrecht, DE) overnight (30 °C, 1000 rpm).

Afterward, 800 μl buffered methanol‐complex medium with yeast extract (BMMY) containing 10 μM hemin chloride (dissolved in methanol, final concentration 0.5%) were added. The cells were incubated for another 72 h (25 °C, 1000 rpm) with the addition of 0.5% v/v methanol every 24 h. Cells were centrifuged (5 min at 4000 rpm and 4 °C) and the volumetric activity of the cell free supernatant toward ABTS was measured as described below. *K. phaffii* transformants showing the highest volumetric activity in the prescreening were grown in 50 mL BMGY at 30 °C and 200 rpm for 16–20 h. Afterward, 200 mL BMMY medium supplemented with 10 μM hemin was inoculated from the preculture to an OD_600_ of 1 (cells were previously washed with 0.9% sodium chloride). Expression was conducted at 25 °C and 200 rpm for 72 h and 0.5% v/v methanol was added every 24 h. OD_600_ and volumetric activity toward ABTS (see below) were measured daily. After expression, cell free supernatant was concentrated and rebuffered in 50 mM sodium phosphate buffer at pH 7.0 with 2 mM MgCl_2_ using the ultrafiltration technique in an Amicon (Merck Millipore, Massachusetts, USA) stirring cell equipped with an ultrafiltration disc with a pore size of 10 kDa. For storage, 10% of glycerol was added and samples were stored at 4 °C.

##### Fed‐Batch Fermentation and Purification of the Variant A186F

*Abr*UPO_A186F variant was produced by fed‐batch fermentation of *K. phaffii*::pPICZαA_af‐*Abr*UPO_A186F. Basal salt medium (per 1 L: 0.47 g CaSO_4_ × 2 H_2_O, 8 mL H_3_PO_4_ (85%), 9.1 g K_2_SO_4_, 4.2 g KOH, 3.66 g MgSO_4_, 43.5 g glycerol (100%), supplemented with 0.87 mg biotin, 4.35 mL *Pichia* trace metals (per 1 L of PTM_1_ solution: 6 g CuSO_4_ × 5 H_2_O, 0.08 g NaI, 3 g MnSO_4_.*x* H_2_O, 0.5 g CoCl_2_, 20 g ZnCl_2_, 0.02 g H_3_BO_3_, 0.2 g Na_2_Mo_4_ × 2 H_2_O, 65 g FeSO_4_ × 7 H_2_O, 0.2 g biotin, 5 mL H_2_SO_4_) was inoculated to an OD_600_ of 0.5 in a 7.5 L bioreactor (Infors, Bottmingen, Switzerland). Fed‐batch fermentation was performed as described previously.^[^ ^9^ ^]^ The cells were harvested after 8 days by centrifugation (11 325 × *g*, 4 °C, 20 min), and the culture broth was concentrated and rebuffered by tangential flow filtration using three membrane cassettes with a cutoff value of 10 kDa and 50 mM sodium phosphate buffer pH 7.0 with 2 mM MgCl_2_. Afterward, *Abr*UPO_A186F was purified by HIC on an XK16/20 column with Butyl Sepharose HP medium (20 mL, GE Healthcare, Chicago, USA) using an ÄKTApurifier fast protein liquid cghromatography (FPLC) system (GE Healthcare, Chicago, USA) as described previously.^[^ ^9^ ^]^

##### Estimation of Enzyme Concentration

The enzyme concentration for the calculation of specific activities was determined using the Bradford assay. For conversion of substituted benzenes concentration of purified *Abr*UPO was determined by measuring the CO‐difference spectrum using the extinction coefficient *ε*_445_ = 130 000 M^−1^ cm^−1^ of *Abr*UPO.^[^ ^9^ ^]^

##### Determination of Peroxidase and Peroxygenase Activity

Peroxidase activity was determined in a total volume of 200 μl at 25 °C with 5 mM ABTS (*ε*_420_ = 36 000 M^−1^ cm^−1^) as substrate in McIlvaine buffer pH 4.4 with 1.2 mM H_2_O_2_ as co‐substrate. A volume of 20 μl of enzyme solution was mixed with 140 μl buffer and 20 μl substrate.^[^ ^20^ ^]^ Measurements were started by adding 20 μl of 12 mM H_2_O_2_ solution and the change in absorbance at 420 nm was followed in a photometer. Peroxygenase activity toward NBD (*ε*_425_ = 9,700 M^−1^ cm^−1^) was measured in a total volume of 200 μl at 25 °C. The reaction mixture contained 1 mM NBD and 10% v/v acetonitrile in 50 mM sodium phosphate buffer pH 7.0 supplemented with 2 mM MgCl_2_.^[^ ^21^ ^]^ Measurements were started by adding 20 μl 12 mM H_2_O_2_ and followed at 425 nm on a spectrophotometer. All measurements were conducted in triplicate.

##### Oxidation of Substituted Benzenes

Reactions were conducted in 500 μl volume in 1.5 mL reaction tubes at 25 °C and 600 rpm. The standard reaction mixture contained 1.3 μM UPO, 4 mM H_2_O_2_, 1 mM substrate (**1a**‐**5a**) (dissolved in acetonitrile), and 8 mM ascorbic acid in 50 mM sodium phosphate buffer pH 7.0 with 2 mM MgCl_2_, at a final acetonitrile concentration of 5% v/v. Unless otherwise stated, reactions were extracted with 500 μl ethyl acetate after 180 min. And, 500 μM 1‐dodecanol was used as internal standard. Samples were analyzed by gas chromatography with mass spectrometry detector (GC/MS) measurements.

For selected substrates, ethylbenzene **1a**, propylbenzene **2a**, and butylbenzene **3a** product quantification was done in reactions with either 1 μM *Abr*UPO or A186F. Reactions were conducted in a total volume of 500 μl in 50 mM sodium phosphate buffer pH 7.0 with 2 mM MgCl_2_ and 20 mM ascorbic acid for 180 min at 25 °C and 600 rpm. In total 7.21 mM **1a**, 6.76 mM **2a** or 6.43 mM **3a** were used at a final acetonitrile concentration of 10% v/v. And, 3.33 mM H_2_O_2_ was used to start the reaction and was added in the same concentration in a batch‐fed every 60 min to a total concentration of 10 mM. Samples were extracted with 500 μl ethyl acetate and analyzed *via* achiral or chiral gas chromatography with flame ionisation dyetector (GC/FID) measurements. 1‐Dodecane was used as internal standard. All measurements were conducted in triplicate.

##### GC/MS Analysis

Samples (0.5 μL) were injected to a GC/MS instrument (GC/MS‐QP2010 plus, Shimadzu, Germany) equipped with FS‐Supreme5 column (30 m × 0.25 mm × 0.25 μm, Chromatographie Service GmbH, Germany). The temperature protocols are shown in Table S3, Supporting Information. Conversions were calculated based on substrate depletion (control was set to 100%) and product distributions based on relative peak areas (%) in relation to the internal standard. Substrates and products were identified by comparison with authentic standards or with mass spectrometric data in the NIST20 database according to Schmitz et al.^[^ ^9^ ^]^

##### GC/FID Analysis

Product quantification was done on a Shimadzu GS‐2010 Pro plus/FID equipped with either an achiral Agilent CP‐Sil 5CB column (25 m × 0.25 mm × 1.2 μm) or a chiral Agilent CP Chirasil Dex CB column (25 m × 0.32 mm × 0.25 μm). Temperature protocols for the chiral column are shown in Table S4, Supporting Information, and for the achiral column in Table S5, Supporting Information. Concentrations were calculated based on calibration curves with 1‐dodecane as internal standard. Retention times can be found in Table S6, Supporting Information. Because an authentic standard for **2d**, **2e**, **3d**, **3e**, and **3f** was not available, the response factor of **1d**, **1e**, and **1f** was used to calculate the respective concentrations. Calibration curves are shown in Figure S9–S23, Supporting Information.

##### Homology Modelling and Molecular Docking Analysis

The initial structure of ethylbenzene **1a** was created with the embedded builder function in PyMOL.^[^ ^22^ ^]^ To optimize the homology models from AlphaFold2 and derive the general Amber force field parameters, quantum mechanics calculations were performed at the HF6‐31G* level using Gaussian 09.^[^ ^23^ ^]^ The partial charges were determined through restrained electrostatic potential calculations using the Antechamber module in AmberTools20.^[^ ^24^ ^]^ The ACPYPE tool was employed to generate the GROMACS input files.^[^ ^25^ ^]^ To obtain an initial binding pose for docking simulations of the UPO–substrate complexes, molecular docking using AutoDock Vina was conducted.^[^ ^26^ ^]^ The geometry‐optimized structure generated during the parametrization process was used. The ligand was docked into the active site of each UPO variant, which was defined by a cubic grid with an edge length of 12 Å centered around the iron atom of the heme using AutoDock Tools.^[^ ^27^ ^]^

## Conflict of Interest

The authors declare no conflict of interest.

## Supporting information

Supplementary Material

## Data Availability

The data that support the findings of this study are available from the corresponding author upon reasonable request.
