# Supplementary material for: Identification of Key Active‐Site Positions Controlling the Chemoselectivity of Aspergillus Brasiliensis Unspecific Peroxygenase
Source: Chembiochem. 2025 May 6;26(10):e202500181. doi: 10.1002/cbic.202500181 (PMC12117455; doi:10.1002/cbic.202500181)
Supplement: Supplementary file 1 — Supplementary Material [file CBIC-26-e202500181-s001.pdf]

Supplementary information

Identification of key active site positions controlling the chemoselectivity of *Aspergillus brasiliensis* unspecific peroxygenase

Fabian Schmitz[a], Maike Hoffrogge[a], Katja Koschorreck[a], Yasuhisa Fukuta[a, b], Alessandra Raffaele[a], Florian Tieves[a], Thomas Hilberath[c], Frank Hollmann[c], Vlada B. Urlacher*[a]

[a] F. Schmitz, M. Hoffrogge, Assoc. Prof. Dr. Y. Fukuta, A. Raffaele, Dr. K. Koschorreck, Dr. F. Tieves, Prof. Dr. VB. Urlacher*
Institute of Biochemistry
Heinrich-Heine-Universität
Universitätsstraße 1, 40225 Düsseldorf, Germany
E-mail: Vlada.Urlacher@uni-duesseldorf.de

[b] Assoc. Prof. Dr. Y. Fukuta
Faculty of Agriculture
Kindai University
3327-204 Naka-machi, Nara 631-8505, Japan

[c] Dr. T. Hilberath, Prof. Dr. F. Hollmann
Department of Biotechnology
Delft University of Technology
Van der Maasweg 9, 2629 Hz Delft, The Netherlands

Improvement of the expression level by exchanging the native signal peptide of *Abr*UPO

Due to the limited availability of unspecific peroxygenases (UPOs) expressed by recombinant hosts such as *Komagataella phaffii*, it has always been an approach to optimize/change the wild-type native signal peptides. For example, 4 mutations in N-terminal secretion signal from *Saccharomyces cerevisiae* alpha-factor (further referred to as α-factor signal sequence) led to a 27-fold increase in the functional expression of an UPO from *Agrocybe aegerita* (*Aae*UPO) leading to the improved form PaDa-I.[26a] Further, Püllmann *et al.* developed a modular Golden Gate UPO expression system which allows the simple and rapid exchange of different signal peptides.[26b] With a yield of 740 mg l^{-1}, *Abr*UPO shows the highest expression level of a wild-type UPO expressed in *K. phaffii* so far.[10] To further improve the secretion level we tested five different signal peptides. Among them, the α-factor signal sequence, which has been excellently established for *K. phaffii*, the evolved α-factor signal sequence of PaDa-I, the Ost1-α-factor chimeric signal sequence, which is a fusion of the S*. cerevisiae* Ost1 signal and the α-factor pro-region, the native signal peptide of *Galerina marginata* UPO (*Gma*-SP), and the signal peptide of yeast K1-killer toxin.[26a-e] These signal peptides have been reported to improve the functional expression of different proteins/enzymes. For example, the use of the *Gma*-SP with PaDa-I led to a 2.5-fold further improvement compared to evolved α-factor signal sequence.[26b] Also the Ost1-α-factor chimeric signal sequence could improve the functional expression of a model oligomer, E2-Crimson, 20-fold.[26c] As shown in **Fig. S1** the α-factor signal peptide led to a ~60% higher relative volumetric activity of *Abr*UPO compared to the native signal peptide while the evolved α-factor led to a 50% lower activity. For the signal sequences Ost1-α-factor, the K1-killer toxin and the *Gma*-SP no much difference was observed. This shows that a beneficial effect on the functional expression is highly dependent on the combination of a signal peptide and the protein of interest. Our results allow us to suggest, that no much of an improvement for *Abr*UPO can be achieved by replacing the signal peptide.

During mutant screening, to maximize enzyme expression, all mutants were expressed using the α factor signal sequence due to its 60% enhancement over the native signal peptide.

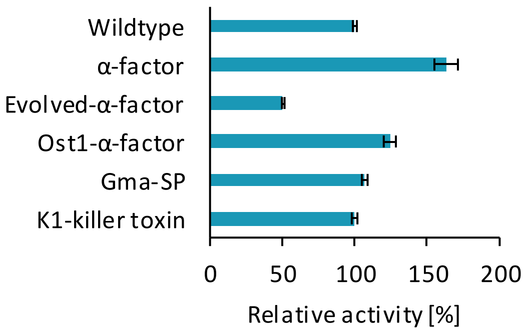

Figure S1: Relative volumetric activity towards ABTS for *Abr*UPO secreted by *K. phaffii* using different signal peptides. Activity was calculated relative to the activity of the enzyme secreted with native signal peptide of *Abr*UPO (Wildtype). Error bars were determined based on standard deviation with sample size n=3.

Table S1: Product distribution in conversion of **1a**, **2a** and **3a** by purified *Abr*UPO and variant A186F. Reactions were conducted in 50 mM sodium phosphate buffer pH 7.0 with 2 mM $MgCl_2$, 7.21 mM **1a**, 6.76 mM **2a** or 6.43 mM **3a** as substrate. 3.33 mM hydrogen peroxide added every 60 min (10 mM final concentration). 1 µM enzyme, 20 mM ascorbate at 25 °C and 600 rpm for 180 min. Product quantification via an internal calibration was done by GC/FID.

		Product distribution [%]				
		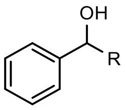	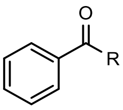	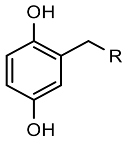	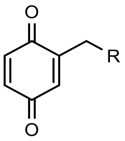	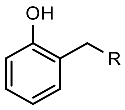
		**b**	**c**	**d**	**e**	**f**
**wild-type**	R=CH_3	24 (24% ee_S)	19	51	1	5
	R=CH_2-CH_3	35 (60% ee_S)	15	45	1	3
	R=CH_2-CH_2-CH_3	31 (38% ee_R)	31	36	0	1
**A186F**	R=CH_3	52 (19% ee_S)	44	3	0	0
	R=CH_2-CH_3	22 (71% ee_S)	77	1	0	0
	R=CH_2-CH_2-CH_3	15 (61% ee_R)	84	1	0	0

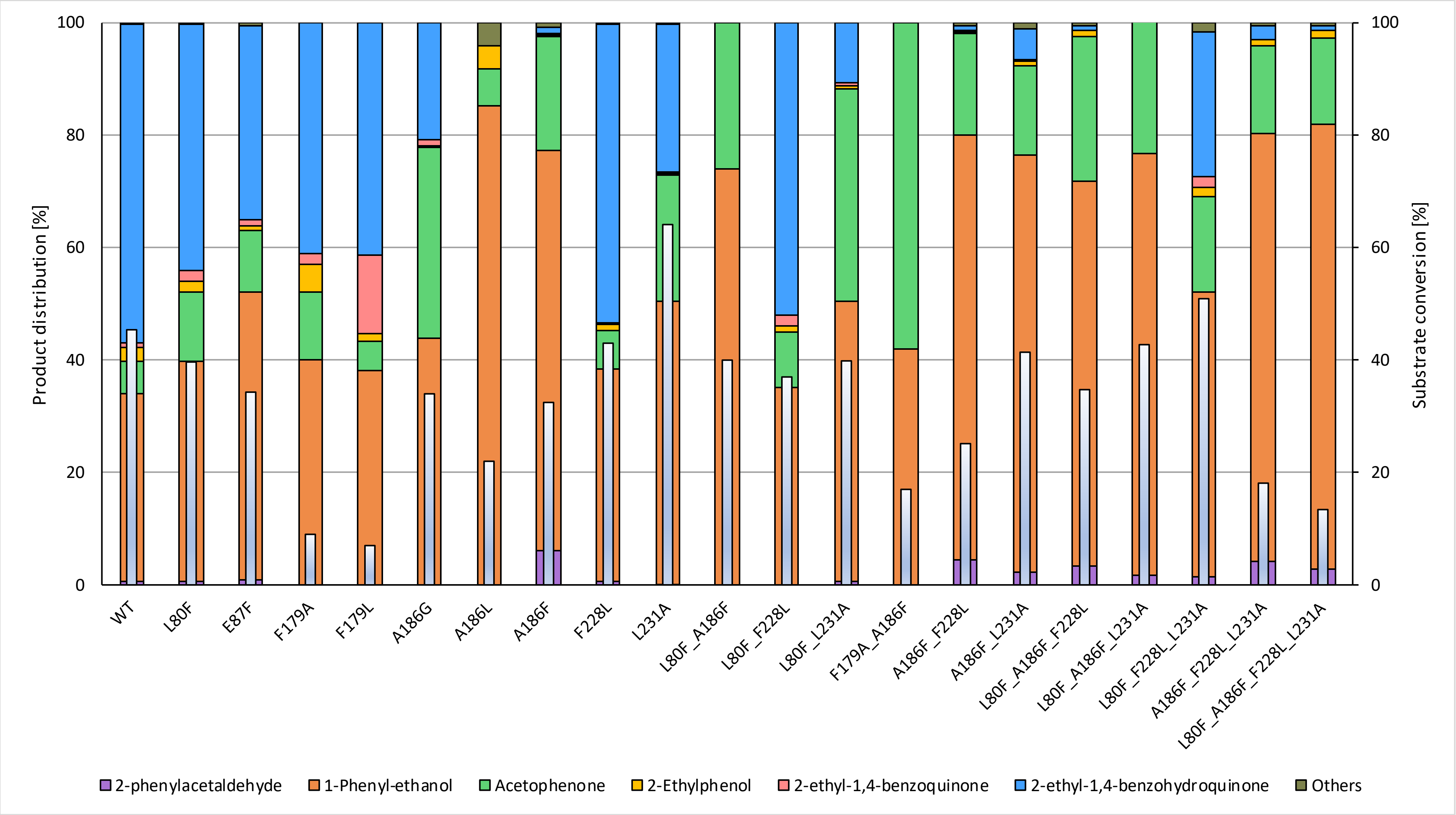

Figure S2: Substrate conversion (grey bars) and product distribution for ethylbenzene **1a**. Reactions were conducted in 50 mM sodium phosphate buffer pH 7.0 with 2 mM $MgCl_2$, 1 mM substrate, 4 mM hydrogen peroxide, 1 µM *Abr*UPO, 8 mM ascorbic acid at 25 °C and 600 rpm for 180 min.

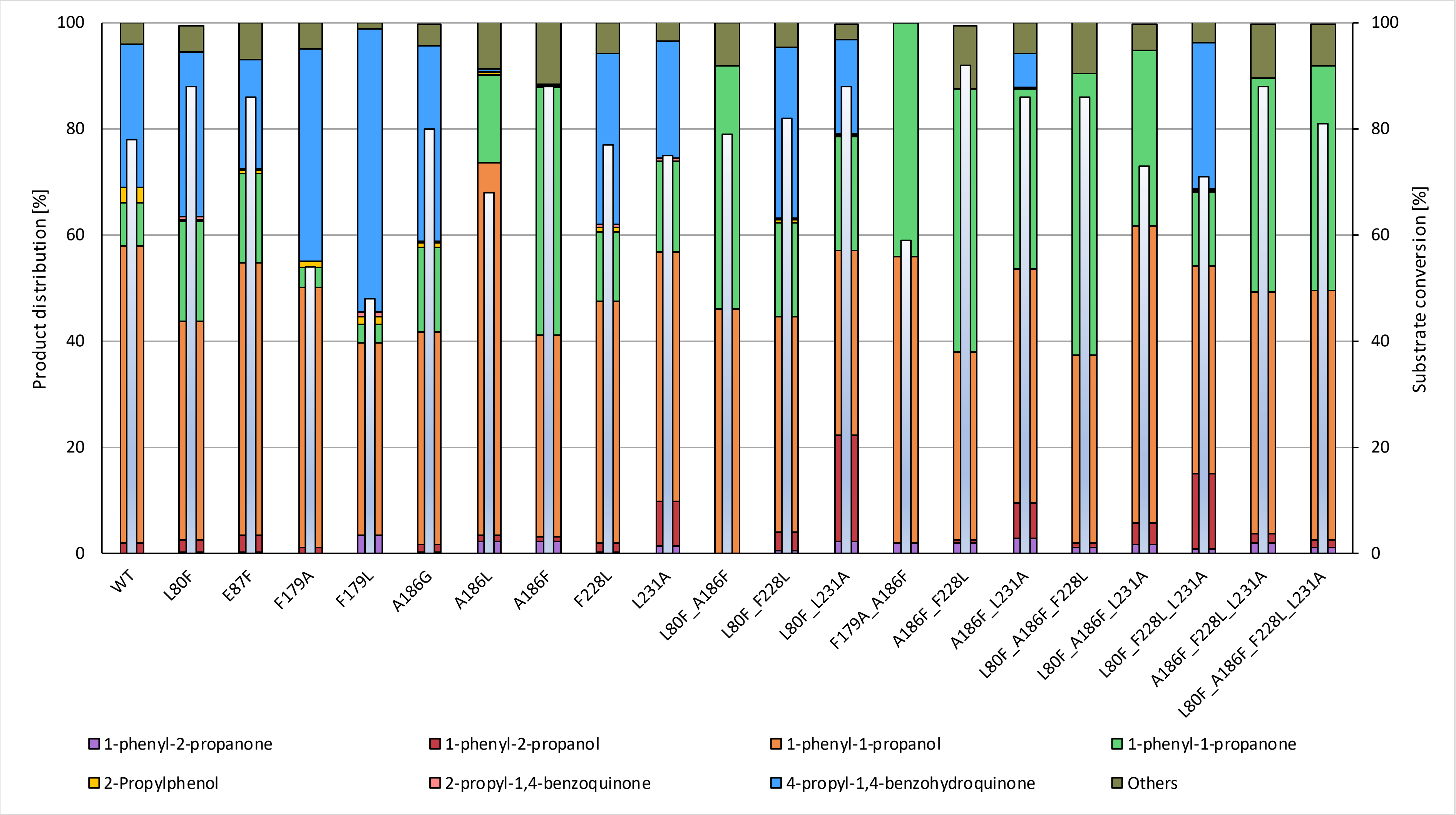

Figure S3: Substrate conversion (grey bars) and product distribution for propylbenzene **2a**. Reactions were conducted in 50 mM sodium phosphate buffer pH 7.0 with 2 mM $MgCl_2$, 1 mM substrate, 4 mM hydrogen peroxide, 1 µM *Abr*UPO, 8 mM ascorbic acid at 25 °C and 600 rpm for 180 min.

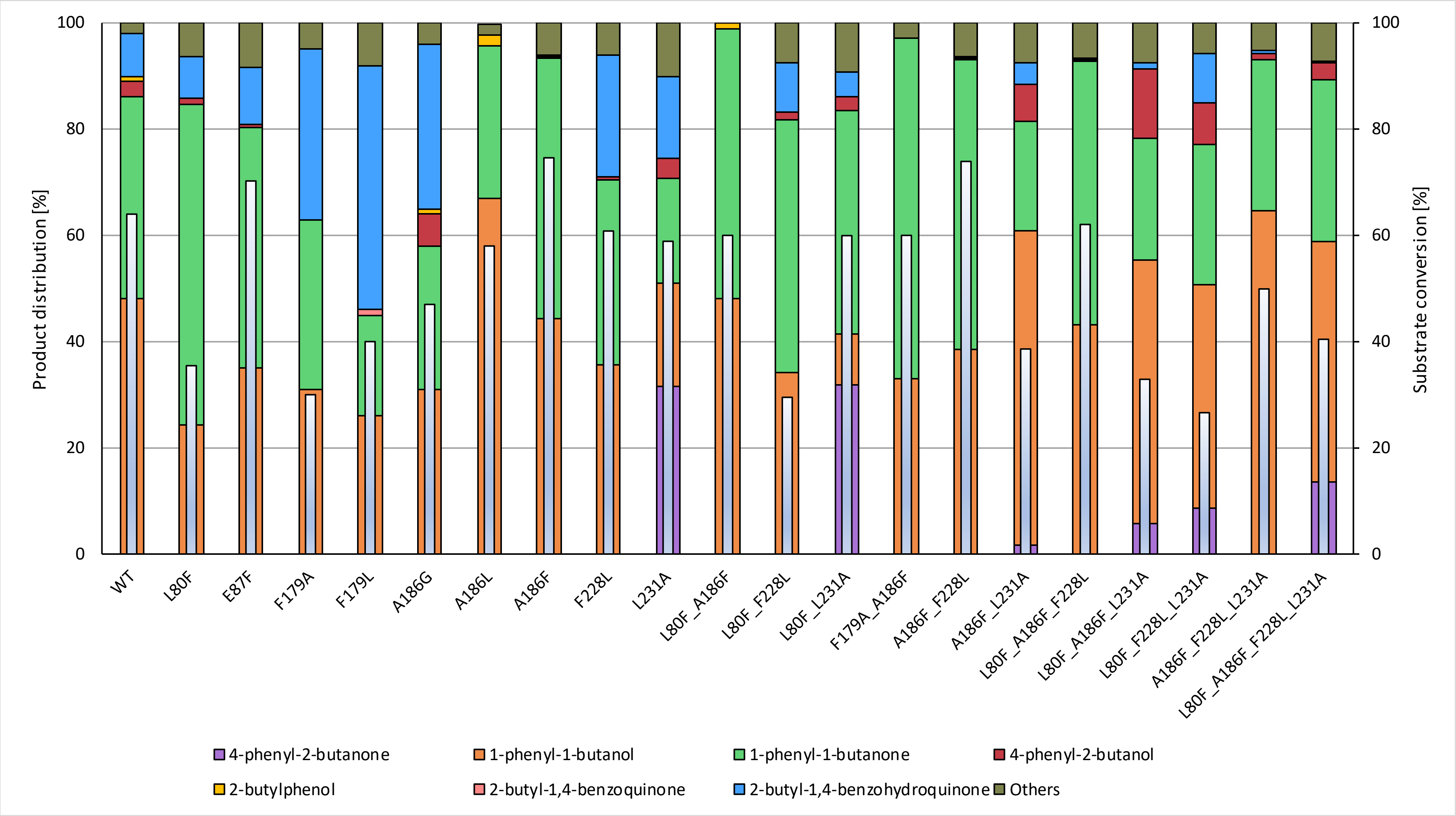

Figure S4: Substrate conversion (grey bars) and product distribution for butylbenzene **3a**. Reactions were conducted in 50 mM sodium phosphate buffer pH 7.0 with 2 mM $MgCl_2$, 1 mM substrate, 4 mM hydrogen peroxide, 1 µM *Abr*UPO, 8 mM ascorbic acid at 25 °C and 600 rpm for 180 min.

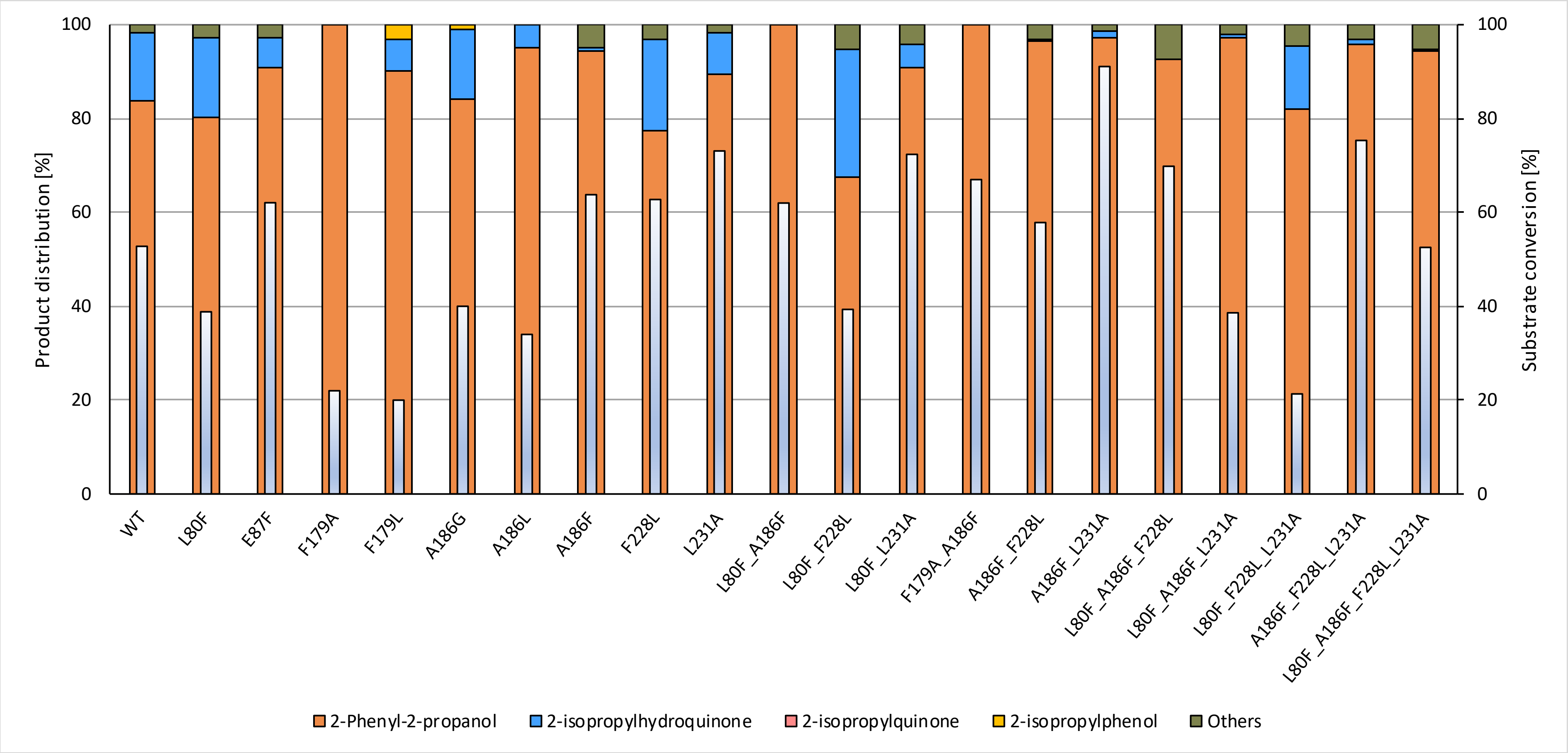

Figure S5: Substrate conversion (grey bars) and product distribution for cumene **4a**. Reactions were conducted in 50 mM sodium phosphate buffer pH 7.0 with 2 mM $MgCl_2$, 1 mM substrate, 4 mM hydrogen peroxide, 1 µM *Abr*UPO, 8 mM ascorbic acid at 25 °C and 600 rpm for 180 min.

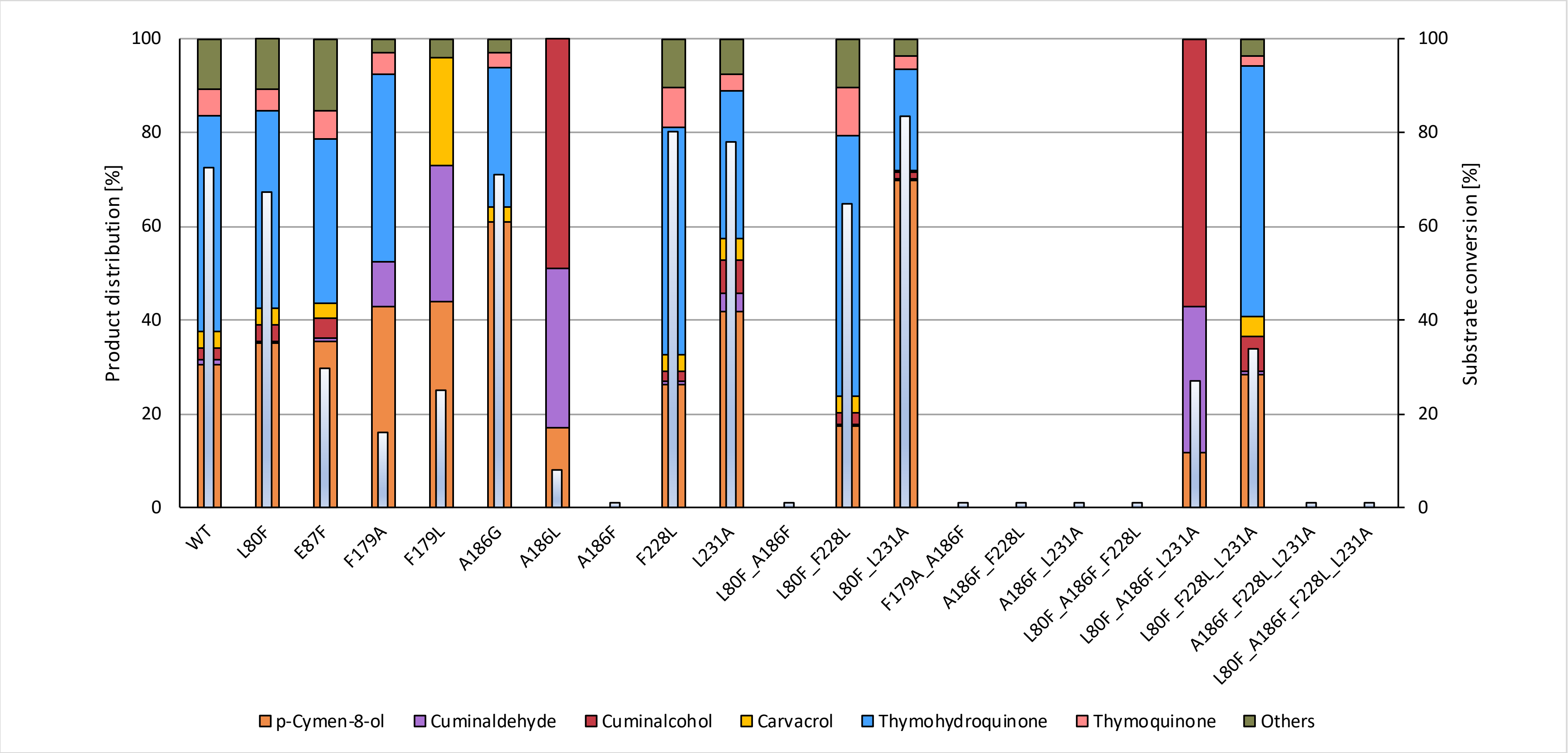

Figure S6: Substrate conversion (grey bars) and product distribution for *p*-cymene **5a**. Reactions were conducted in 50 mM sodium phosphate buffer pH 7.0 with 2 mM $MgCl_2$, 1 mM substrate, 4 mM hydrogen peroxide, 1 µM *Abr*UPO, 8 mM ascorbic acid at 25 °C and 600 rpm for 180 min.

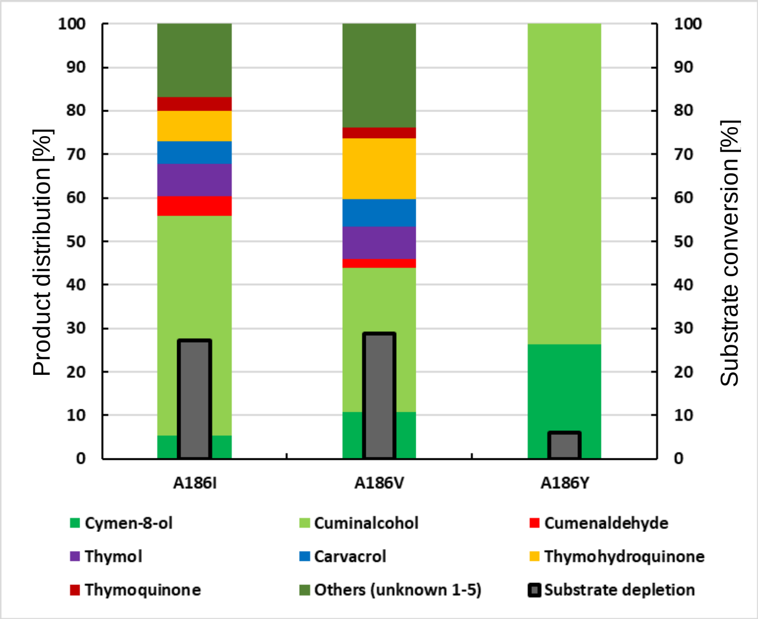

Figure S7: Substrate conversion and product distribution in conversion of *p*-cymene **5a** by different *Abr*UPO variants with amino acid exchanges at position 186. Reactions were conducted in 50 mM sodium phosphate buffer pH 7.0 with 2 mM $MgCl_2$, 1 mM substrate, 4 mM H_2O_2, 1 µM enzyme, 8 mM ascorbic acid at 25 °C and 600 rpm for 180 min.

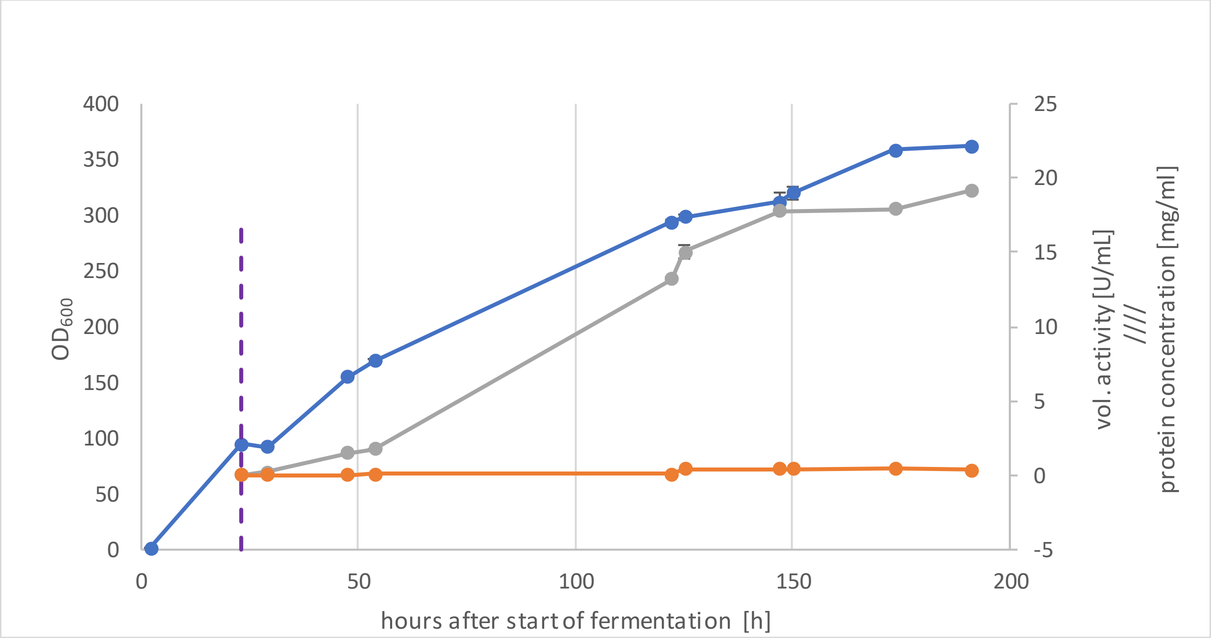

Figure S8: Fed-batch cultivation of recombinant *K. phaffii* X-33 in a 7.5 l bioreactor to produce *Abr*UPO_A187F. Blue: OD_{600}, gray: volumetric activity [U/ml], orange: protein concentration [g/l]. Error bars were determined based on standard deviation with sample size n=3.

Table S2: List of used oligonucleotides.

**Name**	**Sequence in 5'-3' direction**
αf-*Abr*UPO_fw	TAT ACT CGA GAA AAG AGA GGC TGA AGC TTT CCC TCA ACA AGG TGT TCC
Ost1-αf-*Abr*UPO_fw	GAG ATT CGA AAC GAT GAG GCA GGT TTG GTT CTC TTG GAT TGT GGG ATT GTT CCT ATG TTT TTT CAA CGT GTC TTC TGC TGC TCC AGT CAA CAC TAC AAC AGA AG
evαf-*Abr*UPO_fw	TAT ACT CGA GGC CCG AGC CTT CCC TCA ACA AGG TGT TC
*Gma*_fw	GAG ATA TTG CCA ACT CTT GAG ATC AGG GCT TTC CCT CAA CAA GGT GTT CCA CAT CCA TTG
K1_fw	TTT ATC ACG CTA CTT CAT CTA GTC GTC GCT TTC CCT CAA CAA GGT GTT CCA CAT CCA TTG
pPICZA_rev	GCG CTA TTC AGA TCC TCT TC
L80F_fw	GGT ATT GAT GCT GAA **TTC** GCT ACT TAC TTG C
L80F_rev	G CAA GTA AGT AGC **GAA** TTC AGC ATC AAT ACC
E87F_fw	CT TAC TTG CAT CAA **TTC** GCT GTT ACT ACT AAC CC
E87F_rev	GG GTT AGT AGT AAC AGC **GAA** TTG ATG CAA GTA AG
F179A_fw	GAA TTG GGT GAT GCC **GCC** TCT TAT GGT GAA AC
F179A_rev	GTT TCA CCA TAA GAG G**CG G**CA TCA CCC AAT TC
F179L_fw	GAA TTG GGT GAT GCC **TTG** TCT TAT GGT GAA AC
F179L_rev	GTT TCA CCA TAA GA**C AA**G GCA TCA CCC AAT TC
A186G_fw	GGT GAA ACT GCT **GGC** TAC ATT ATT GTT CTT GG
A186G_rev	CCA AGA ACA ATA ATG TA**G CC**A GCA GTT TCA CC
A186L_fw	GGT GAA ACT GCT **CTC** TAC ATT ATT GTT CTT GG
A186L_rev	CCA AGA ACA ATA ATG TA**G AG**A GCA GTT TCA CC
A186F_fw	GGT GAA ACT GCT **TTC** TAC ATT ATT GTT CTT GG
A186F_rev	CC AAG AAC AAT AAT GTA **GAA** AGC AGT TTC ACC
F228L_fw	GA GAA AAC ATT ACC **TTG** GAT GAT CTT AGT AC
F228L_rev	GT ACT AAG ATC ATC **CAA** GGT AAT GTT TTC TC
L231A_fw	C ATT ACC TTC GAT GAT **GCT** AGT ACT ATG TTG
L231A_rev	CAA CAT AGT ACT **AGC** ATC ATC GAA GGT AAT G
F228L_L231A_fw	C ATT ACC TTG GAT GAT **GCT** AGT ACT ATG TTG
F228L_L231A_rev	CAA CAT AGT ACT **AGC** ATC ATC CAA GGT AAT G

Table S3: Temperature profiles for GC/MS analysis on an achiral column for screening application.

Compound	Temperature profile
**1a-3a**	Maintained at 80 °C for 5 min, ramped to 250 °C at 20 °C min^{-1}, held for 2 min
**4a-5a**	Maintained at 100 °C for 1 min, ramped to 150 °C at 5 °C min^{-1}, held for 1 min, ramped to 250 °C at 20 °C min^{-1}, held at 250 °C for 1 min
**6a**	Maintained at 70 °C for 2 min, ramped to 100 °C at 5 °C min^{-1}, ramped to 250 °C at 15 °C min^{-1}, held at 250 °C for 2 min

Table S4: Temperature profiles for GC/MS analysis on a chiral column for quantification application.

Compound	Temperature profile
**1a**	Maintained at 120 °C for 2.60 min, ramped to 135 °C at 15 °C min^{-1}, held for 3.30 min, ramped to 225 °C at 25 °C min^{-1}, held at 225 °C for 1 min
**2a**	Maintained at 90 °C for 3 min, ramped to 125 °C at 5 °C min^{-1}, held for 5 min, ramped to 225 °C at 25 °C min^{-1}, held at 225 °C for 1 min
**3a**	Maintained at 120 °C for 5 min, ramped to 135 °C at 5 °C min^{-1}, held for 8 min, ramped to 225 °C at 25 °C min^{-1}, held at 225 °C for 1 min

Table S5: Temperature profiles for GC/MS analysis on an achiral column for quantification application.

Compound	Temperature profile
**1a-3a**	Maintained at 170 °C for 3 min, ramped to 345 °C at 15 °C min^{-1}, held at 345 °C for 1 min

Table S6: Retention times of **1a**, **2a**, **3a** and their oxidation products on GC/FID.

Compound	Ret. Time [min]	GC column
**1a**	2.37	Chiral
**(*R*)-1b / (*S*)-1b**	6.02 / 6.27	Chiral
**1c**	4.12	Chiral
**1d**	8.43	Achiral
**1e**	5.19	Achiral
**1f**	5.31	Achiral
**2a**	5.00	Chiral
**(*R*)-2b / (*S*)-2b**	15.86 / 16.03	Chiral
**2c**	10.63	Chiral
**2d**	9.36	Achiral
**2e**	6.12	Achiral
**2f**	6.24	Achiral
**3a**	3.68	Chiral
**(*R*)-3b / (*S*)-3b**	12.50 / 12.67	Chiral
**3c**	7.91	Chiral
**3d**	10.39	Achiral
**3e**	7.35	Achiral
**3f**	7.55	Achiral

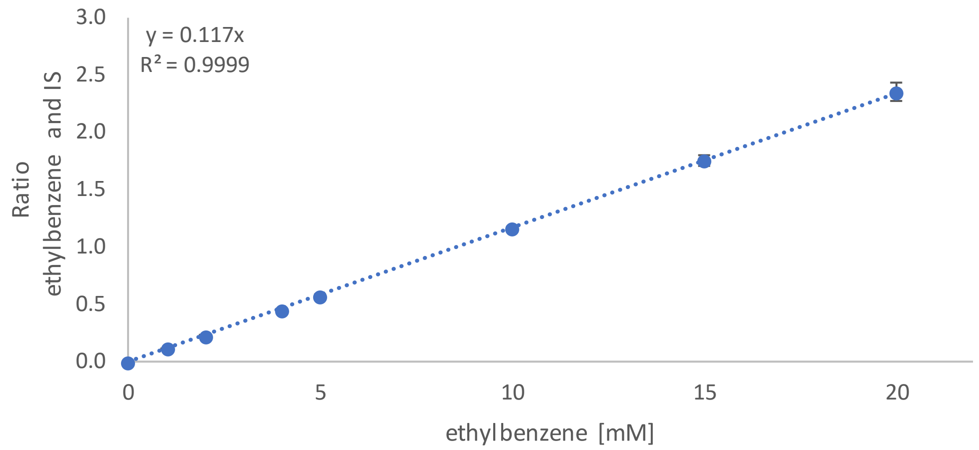

Figure S9: Calibration curve of **1a**. Error bars were determined based on standard deviation with sample size n=3.

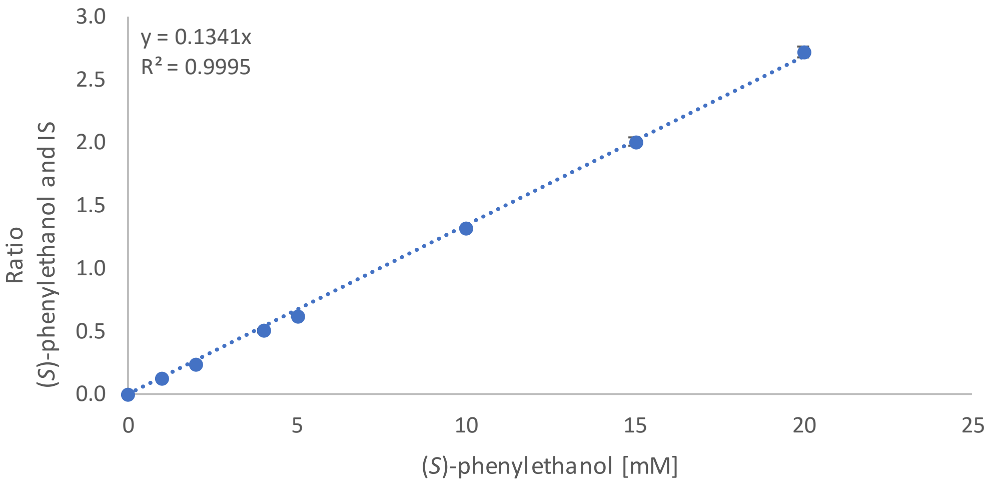

Figure S10: Calibration curve of **(*S*)-1b**. Error bars were determined based on standard deviation with sample size n=3.

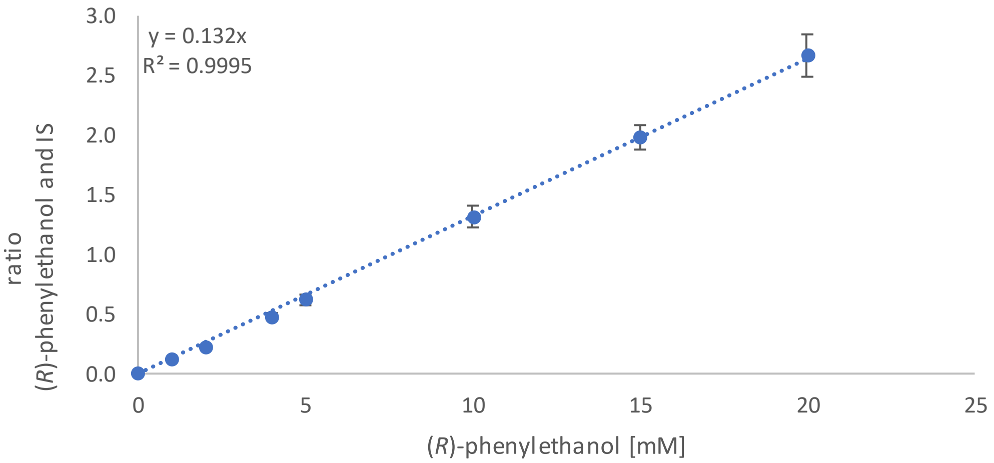

Figure S11: Calibration curve of **(*R*)-1b**. Error bars were determined based on standard deviation with sample size n=3.

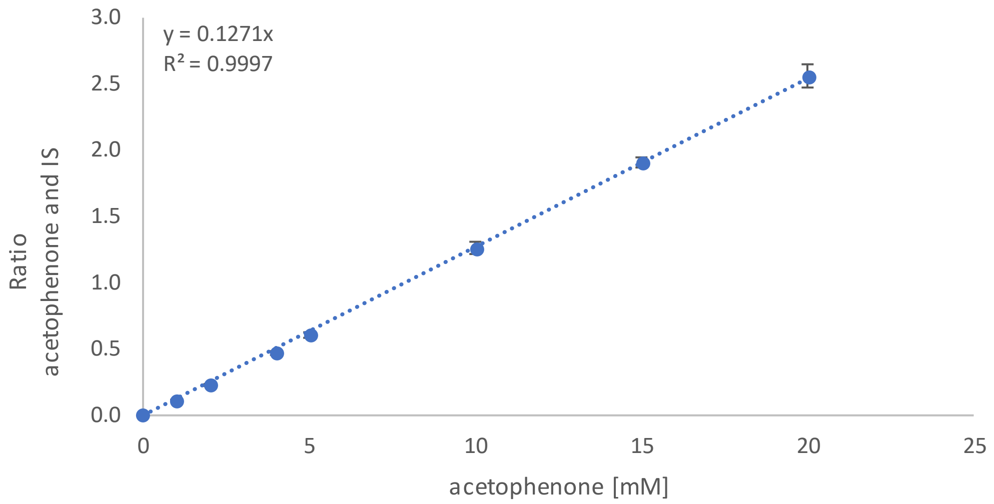

Figure S12: Calibration curve of **1c**. Error bars were determined based on standard deviation with sample size n=3.

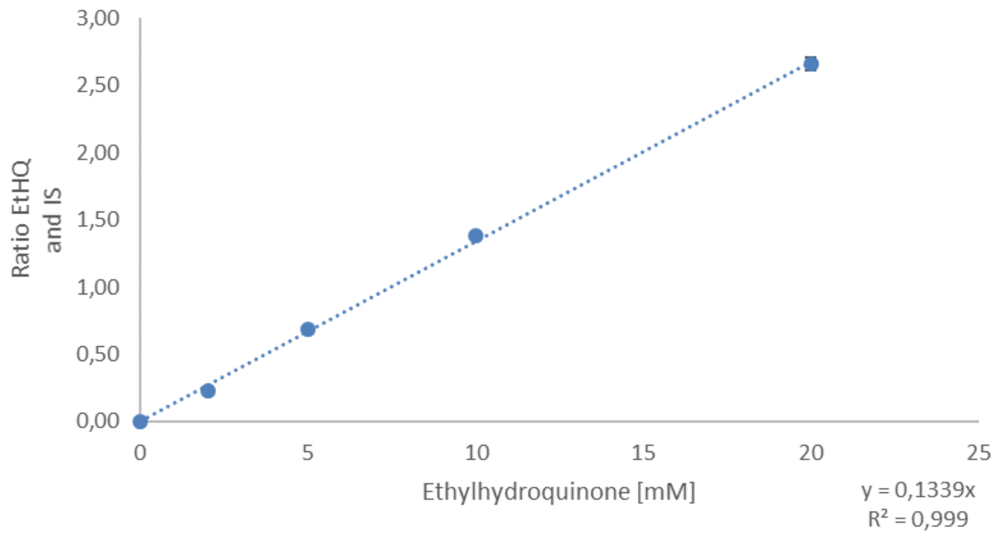

Figure S13: Calibration curve of **1d**. Error bars were determined based on standard deviation with sample size n=3.

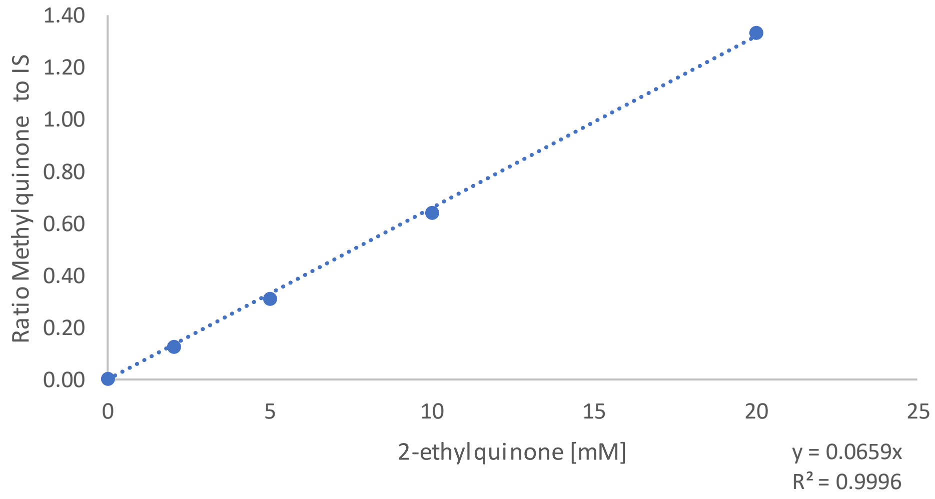

Figure S14: Calibration curve of **1e**. Error bars were determined based on standard deviation with sample size n=3.

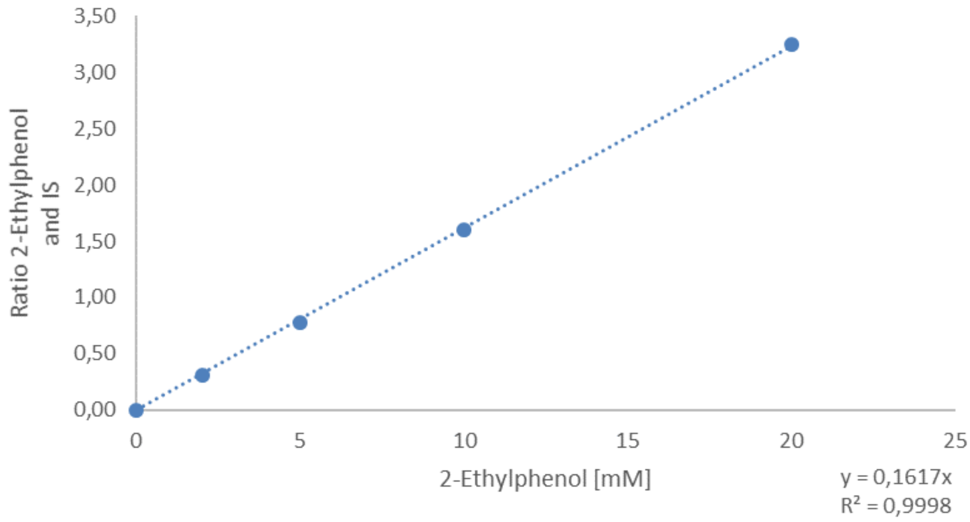

Figure S15: Calibration curve of **1f**. Error bars were determined based on standard deviation with sample size n=3.

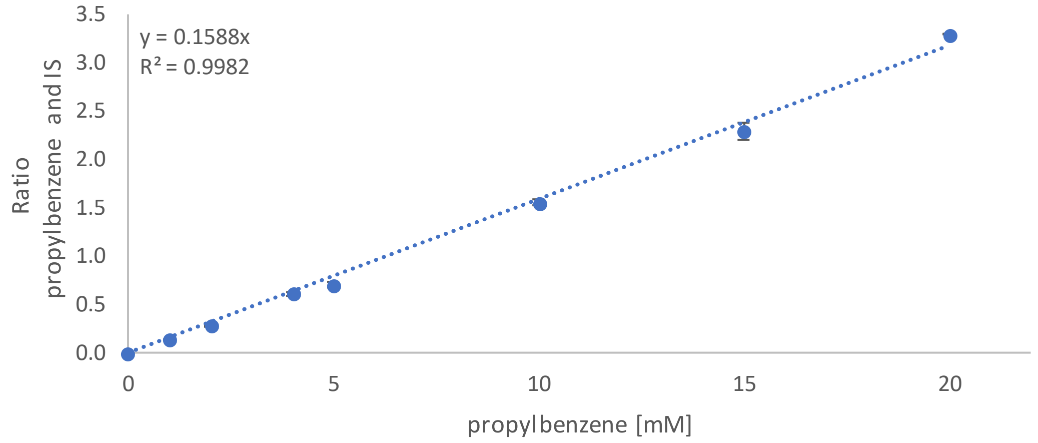

Figure S16: Calibration curve of **2a**. Error bars were determined based on standard deviation with sample size n=3.

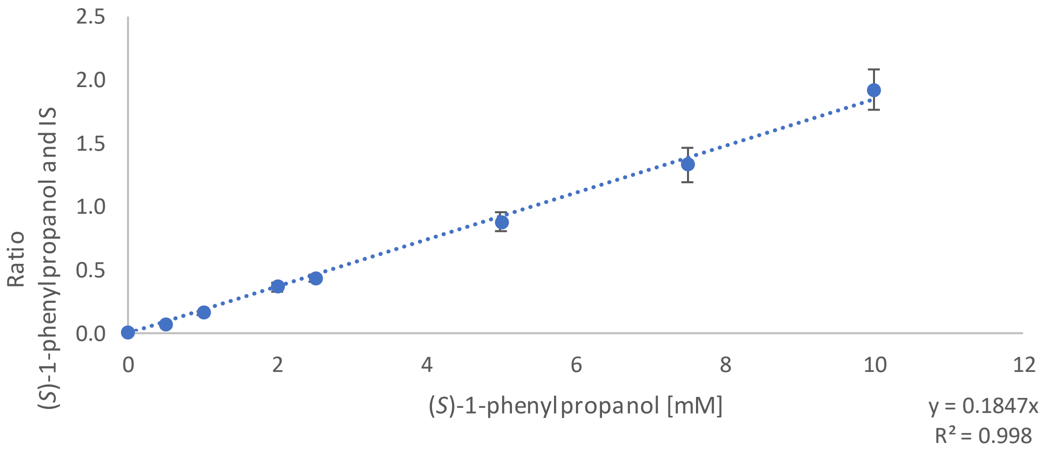

Figure S17: Calibration curve of **(*S*)-2b**. Error bars were determined based on standard deviation with sample size n=3.

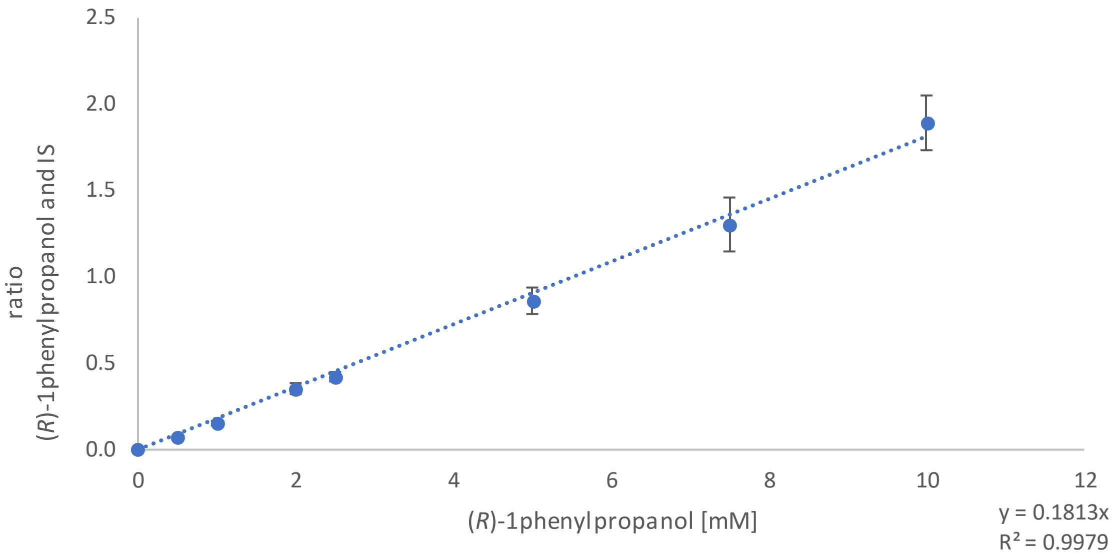

Figure S18: Calibration curve of ***(R)*-2b**. Error bars were determined based on standard deviation with sample size n=3.

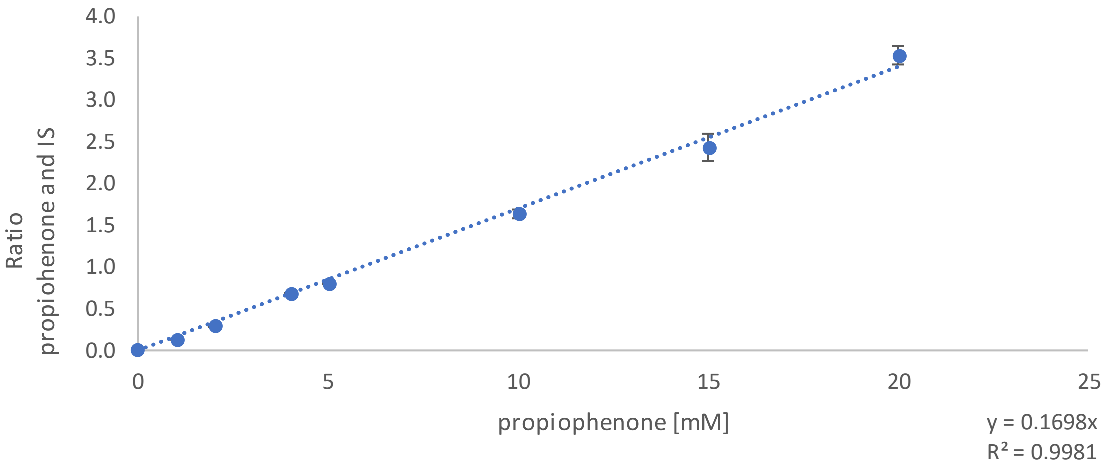

Figure S19: Calibration curve of **2c**. Error bars were determined based on standard deviation with sample size n=3.

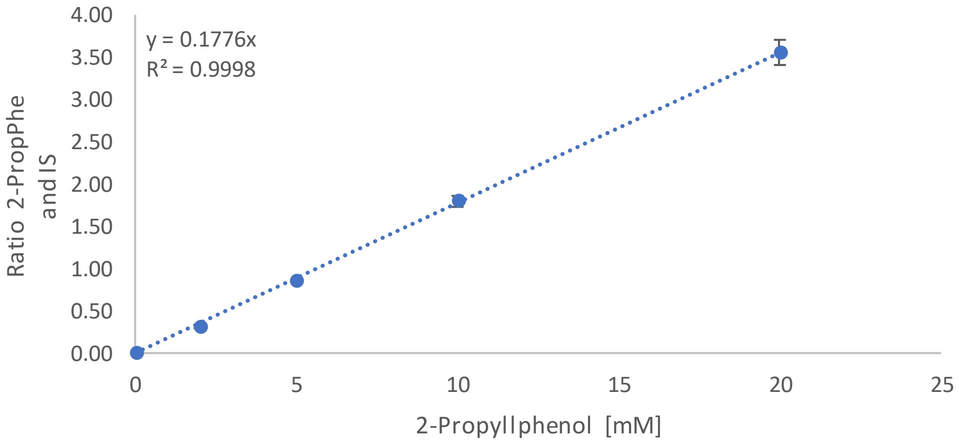

Figure S20: Calibration curve of **2f**. Error bars were determined based on standard deviation with sample size n=3.

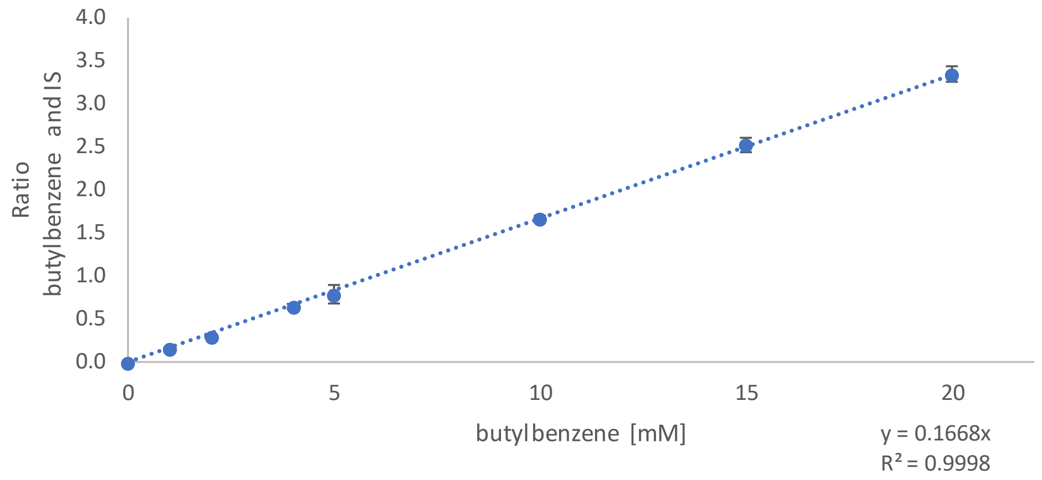

Figure S21: Calibration curve of **3a**. Error bars were determined based on standard deviation with sample size n=3.

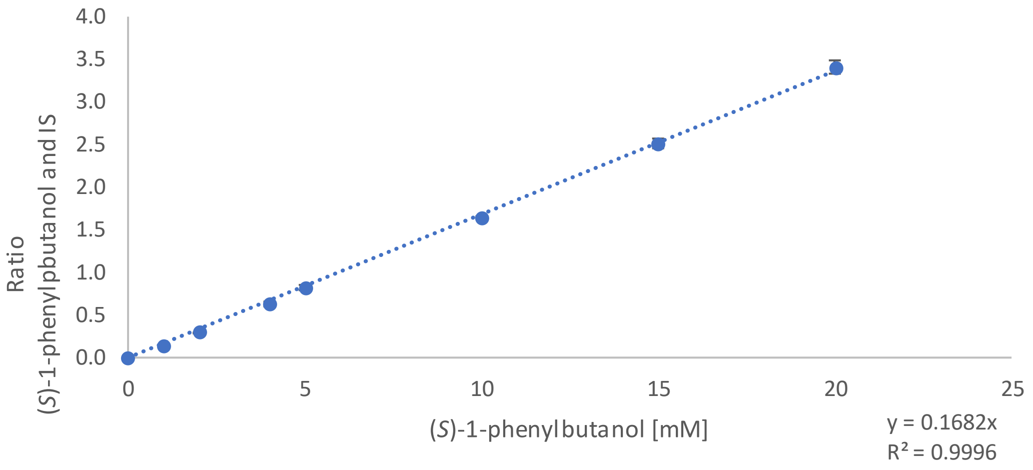

Figure S22: Calibration curve of **(*S*)-3b**. Error bars were determined based on standard deviation with sample size n=3.

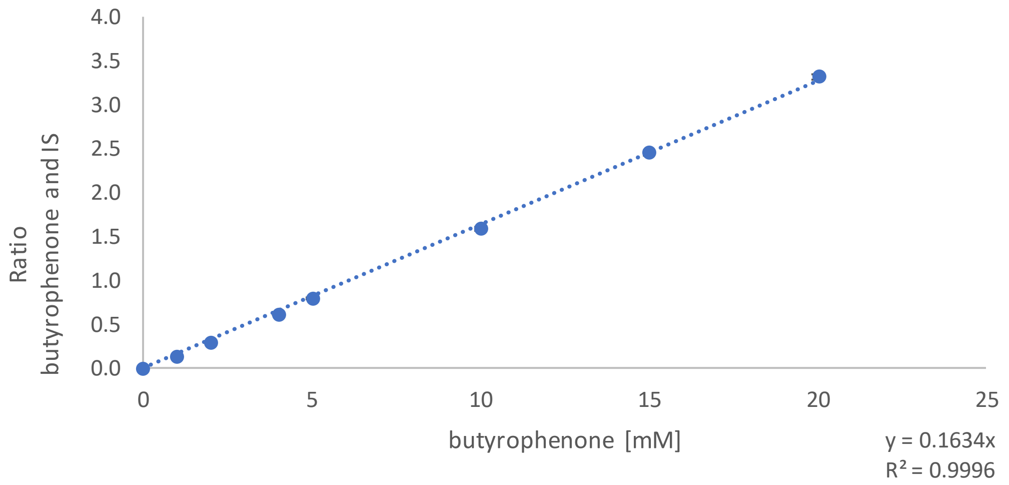

Figure S23: Calibration curve of **3c**. Error bars were determined based on standard deviation with sample size n=3.
